# Research on mechanical properties of a new type of composite floor slab with split joint

**DOI:** 10.1038/s41598-025-96143-y

**Published:** 2025-08-26

**Authors:** Chengquan Wang, Ruiui Qian, Xinquan Wang, Yonggang Shen, Ting Lei, Xi Wu, Lei Han

**Affiliations:** 1 School of Engineering, Hangzhou City University, Hangzhou, 310015 China; 2 Zhejiang Engineering Research Center of Intelligent Urban Infrastructure, Hangzhou, 310015 China; 3 Department of Civil Engineering, Zhejiang University, Hangzhou, 310058 China; 4 Ltd of China Construction Eighth Engineering Division, Zhejiang Construction Co, Hangzhou, 311200 China

**Keywords:** Composite floor slab, New type of split joint, Mechanical properties, Full-scale experiment, Finite element simulation, Theoretical calculation, Civil engineering, Engineering, Theory and computation

## Abstract

At present, the commonly used composite floor of steel bar truss in prefabricated buildings has the problem that the overhanging bent steel bars in the prefabricated floor collide with the reserved steel bars in other prefabricated components, which reduces the subsequent construction efficiency. In order to solve this problem, a new type of composite floor slab with split joints was designed. The bent steel bars are distributed centrally in the middle of the stitching edge of the prefabricated bottom plate, while the ends of the bottom plate are not reinforced, which reduces the contact with the reserved steel bars of other structures.This paper presents the mechanical properties of a new type of composite floor slab with split joint. Based on the loading test of full-scale specimens, finite element numerical simulation and theoretical calculation, the ultimate bearing capacity, elastoplastic properties, mechanical properties of reinforcement at the joints and other basic mechanical properties were investigated. The experiment results shown that the new type of composite floor slab with split joint exhibit better mechanical properties, and the structure of the laminated slabs can better guarantee the force transfer between the precast slabs. The theoretical, experimental, and finite element method (FEM) results are in good agreement, validating the reliability of the proposed composite floor slab design.The research content of this paper provides theoretical support for the application of the new composite floor slab in engineering practice, while its practical applications include improved construction efficiency, enhanced structural integrity, and crack resistance, making it a valuable solution for modern construction.

## Introduction

Composite floor slab is a structural form with good combination of precast and cast-in-situ concrete, which is composed of precast bottom slab and upper composite layer concrete to work together as a whole^1^. Composite floor slab can be divided into many types according to the form of precast bottom slab. Such as ordinary rough prestressed composite floor slab, prefabricated ribbed composite floor slab, reinforced truss composite floor slab, profiled steel plate concrete composite floor slab, etc^2^. Scholars both domestic and foreign have made in-depth research on composite slab. M. V. A. Lima et al.^3^ Combined with the Mazars damage model simulating the stiffness loss in the process of concrete cracking and the classical theory of laminated slab, put forward the prediction model of flexural performance of reinforced concrete floor slab, and established the deformation formula of reinforced concrete floor slab based on the principle of virtual work. Michel Crisinel et al.^4^designed a simplified method to calculate the ultimate bearing capacity of composite floor slabs in combination with the test results, which provides a basis for engineering application. Su Xiaolu^5^ analyzed the cold-rolled ribbed prestressed composite hollow slab with ANSYS, and compared the load-deflection curve, crack and the failure mechanics with the test to verify the rationality of the modeling. Xu Xuekun et al.^6^ created reinforced truss concrete composite floor slab and ribbed reinforced truss concrete composite floor slab with steel fiber recycled concrete as raw materials, established and checked the calculation formulas of their stiffness and bearing capacity, analyzed their crack resistance, and proved that the composite floor slab made of steel fiber recycled concrete has good mechanical performance.

In recent years, research on composite floor systems has focused on improving mechanical properties and performance under various loading conditions. Studies have shown that using alternative materials like recycled aggregates and advanced composites can enhance structural integrity and durability. Innovative design approaches, such as displacement-based methods, have also been explored to optimize seismic performance. For example, Abbas et al.^7^ and Amjad et al.^8^found that using HDPE and EPW in concrete, combined with silica fume, MSF, and NIOP, significantly improved mechanical properties. Qureshi and Warnitchai^9^ and Najam et al.^10^ proposed seismic design techniques to enhance structural performance with lower costs. Joyklad et al.^11^ enhanced brick masonry walls using cement-sand mortar and wire mesh. Yooprasertchai et al.^12^ and Chaiyasarn et al.^13^ showed that steel clamps, hemp fiber ropes, and CFRP wrapping can boost recycled aggregate concrete’s strength and ductility.Khali et al.^14^ investigated the effect of natural fiber rope (HFR) wrapping technology on the compressive performance of cylindrical columns of recycled aggregate concrete (RAC). Through experimental analysis, it was found that HFR wrapping technology significantly improved the compressive strength and ductility of RAC, especially RAC using fire brick aggregates. Suparp et al.^15^ found CFRP wrapping improves RC beams’ ductility and load capacity.However, existing composite floor slabs still face challenges in practical applications. For instance, Madan et al.^16^ and Kanagaraj et al.^17^pointed out that the use of steel reinforcement in composite slabs can lead to corrosion and increased maintenance costs. Furthermore, the integration of sustainable materials like geopolymer concrete (GPC) and expanded polystyrene (EPS) has shown potential in enhancing the performance of composite slabs, but their application in composite floor systems is still limited^18^. Additionally, the use of fiber-reinforced polymer (FRP) sheets as reinforcement in concrete slabs has been explored, but the research on their application in composite floor slabs is still in its infancy^19^.

**Fig. 1:**
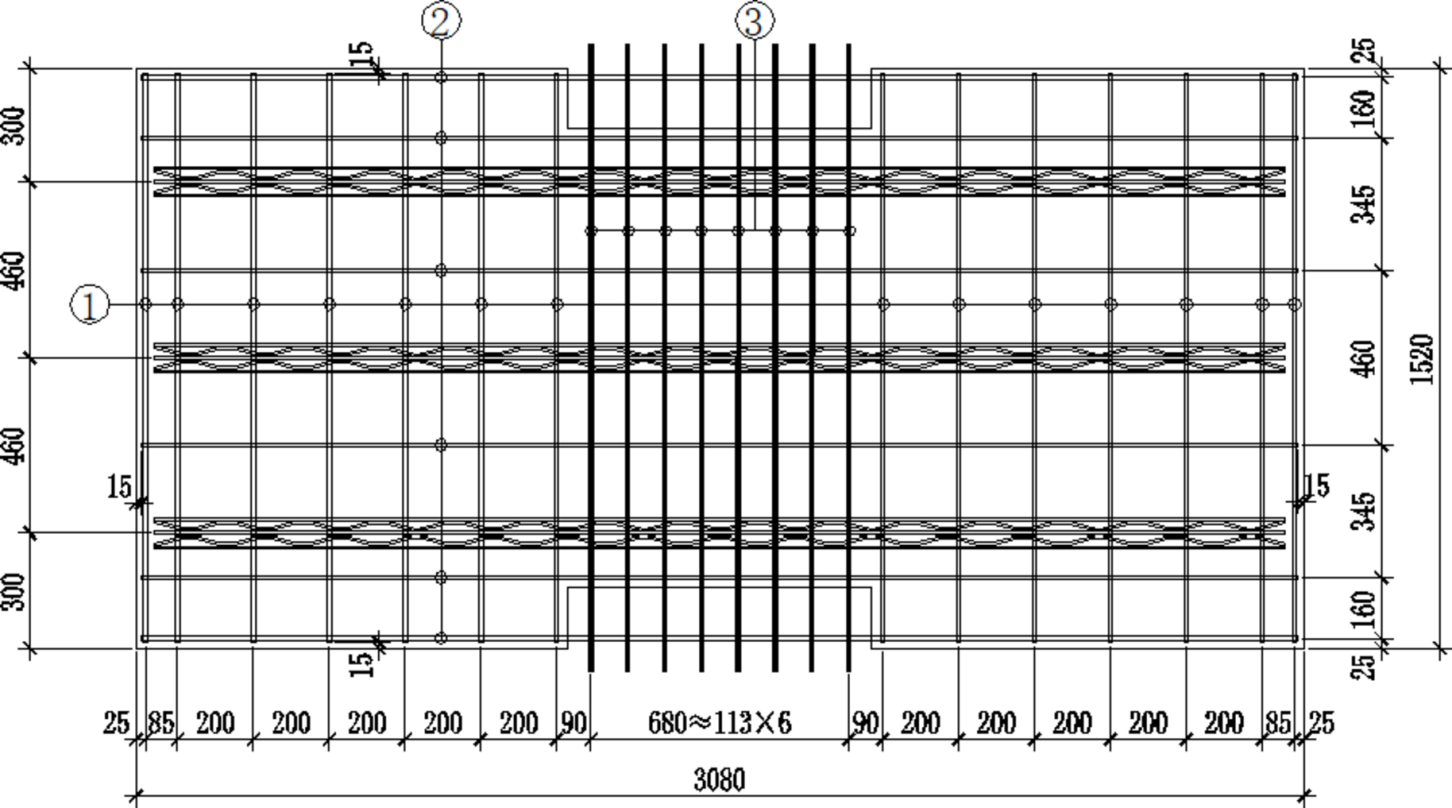
The steel rebar layouts of precast floor.

This study aims to address the challenges associated with existing composite floor slabs, which often suffer from reinforcement collision and compromised structural integrity. To overcome these issues, a new design has been proposed that incorporates sustainable materials and advanced reinforcement techniques. The innovation of this new design lies in its ability to enhance structural integrity while potentially integrating sustainable materials and advanced reinforcement techniques. This study aims to fill the research gap by providing a comprehensive evaluation of the mechanical performance and practical feasibility of the new composite floor slab design.In order to keep integrity of composite slab after splicing, gaps between the plates are reserved. Also, a certain length of bent reinforcement on the splicing edge are considered, and the length of the extended edge is evenly distributed. However, these bent reinforcement are easy to collide with the reserved reinforcement of other prefabricated components, which brings inconvenience to the subsequent construction. Based on this, a new type of composite floor slab with split joint is designed. The bent reinforcement is concentrated in the middle of the splicing edge of the laminated slab to reduce the contact and collision between the reinforcement. The form of its prefabricated bottom slab is shown in Fig. 1. In this paper, two precast bottom plates are spliced and laminated concrete is poured to form a composite slab. The loading test, finite element analysis and theoretical calculation are carried out to study its bearing capacity, deformation characteristics and the mechanical performance of reinforcement at the joint, so as to provide a basis and reference for the practical application of this kind of composite slab.

## Test overview

### Test components

**Fig. 2:**
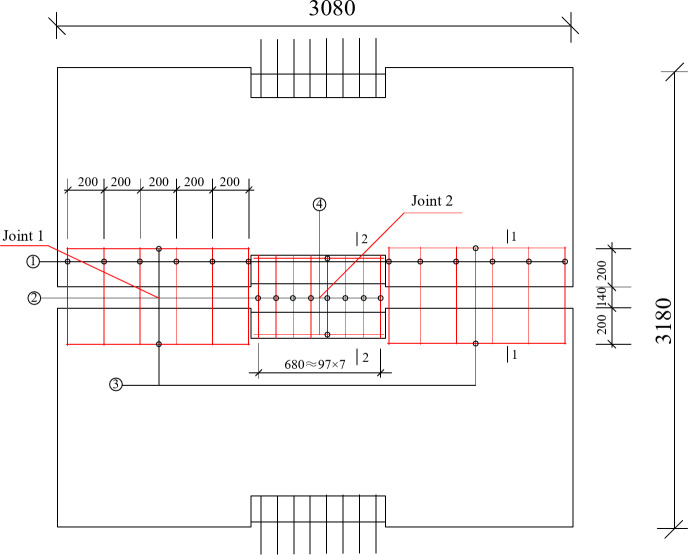
Constructional drawing of composite floor slab with split joint.

The plane size of the composite slab is 3180 mm × 3080 mm, 130 mm thick slab, including 60 mm thick precast bottom slab and 70 mm thick post cast concrete. The spacing between precast bottom slabs is 140 mm. The reinforcement truss bound by precast bottom slab adopts a90 specification. In order to enhance the integrity of the floor slab, additional reinforcement mesh sheets (numbers ① and ③ in the Fig. 2) with a spacing of 97 mm are laid on the surface of the prefabricated floor slab at the joint 1, and the paved reinforcement mesh sheets (numbers ② and ④ in the Fig. 2) with a spacing of 200 mm are arranged at the joint 2.

### Comparison with standard composite slab configurations

The design of the new composite floor slab with split joints represents a departure from traditional composite slab configurations. Standard composite slabs typically feature continuous reinforcement across the joints, which can lead to increased reinforcement congestion and potential construction difficulties, especially in prefabricated systems. In contrast, the new design centralizes the bent-up reinforcement in the middle of the joint, thereby reducing the likelihood of reinforcement collision during assembly. This innovation not only enhances construction efficiency but also maintains structural integrity by ensuring better load transfer between the precast slabs. Additionally, the incorporation of reinforcement mesh sheets at the joints provides enhanced crack resistance, a feature that is often lacking in standard configurations. The new design aims to address these shortcomings while maintaining or improving the mechanical properties of the composite slab.

### Arrangement of strain gauges

**Fig. 3:**
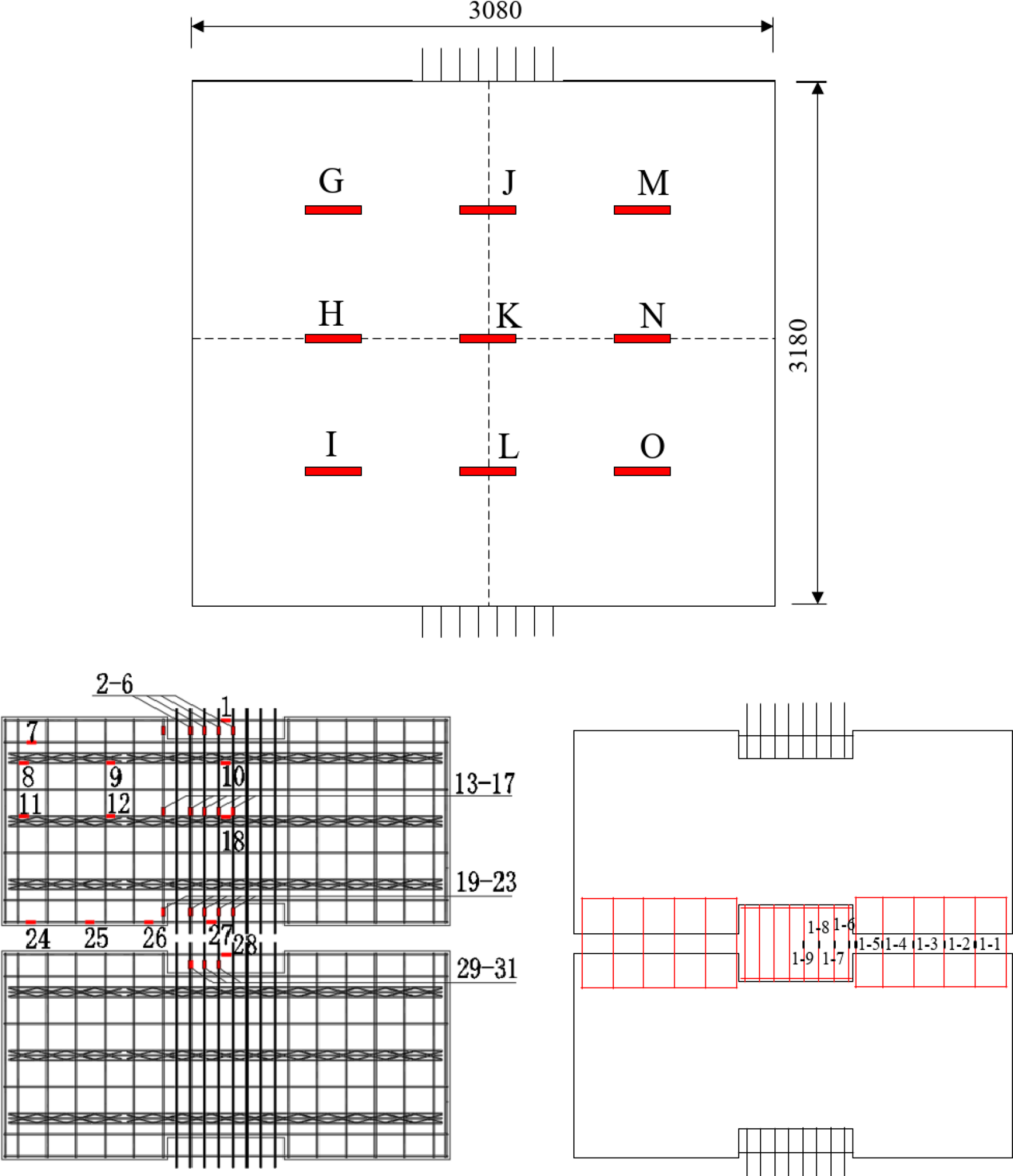
**(a)** Concrete strain gauges at the bottom of precast slab. **(b)** Strain gauges mounted on the individual rebars.

The concrete strain gauge is arranged according to the stress condition of two -way slab. The concrete strain gauge is pasted on the plastic hinge line of two -way slab and the lower surface of the concrete at the joint position to measure the strain change of the bottom concrete during loading. As shown in Fig. 3 (a).

The strain gauge on the reinforcement is mainly arranged on the reinforcement framework of the prefabricated bottom plate and the crack proof reinforcement mesh (paved reinforcement mesh) at the joint of the superimposed floor slab. The specific location is shown in Fig. 3 (b).

The summary of strain gauge locations and expected measurements is shown in Table 1.

**Table 1:** Summary of strain gauge locations and expected measurements.

Component	Strain Gauge Location	Expected Measurement Results
Precast Slab	Concrete lower surface at the joint seam	Strain changes in concrete at the joint seam during loading, used to evaluate crack generation and development
	Center of the precast slab	Strain detection in the center area of the precast slab to evaluate the overall stress condition
	Edge of the precast slab	Strain detection at the edge of the precast slab to evaluate the stress condition of the edge area
Reinforcing Steel Truss	Reinforcing steel truss inside the precast slab	Strain changes in the reinforcing steel truss during loading, focusing on the strain at the joint seam
	Upper chord of the steel truss	Detection of compressive strain in the upper chord to evaluate the stress distribution in the compression zone
	Lower chord of the steel truss	Detection of tensile strain in the lower chord to evaluate the stress distribution in the tension zone
	Web reinforcement of the steel truss	Detection of strain in the web reinforcement to evaluate its stress condition during loading
Crack-Resistant Reinforcement Mesh	Joint seam in the composite layer	Strain changes in reinforcement at the joint seam during loading, used to evaluate crack control effectiveness
	Center of the composite layer	Detection of strain in the center area of the composite layer to evaluate the overall stress condition
	Edge of the composite layer	Detection of strain in the edge area of the composite layer to evaluate the stress condition of the edge area
Connection Reinforcement	Connection reinforcement at the joint seam	Strain changes in connection reinforcement during loading, used to evaluate the load transfer effect at the joint seam
	Distance from connection reinforcement to the center of the slab	Detection of strain in connection reinforcement at different positions to evaluate the stress distribution in the joint seam
Cast-in-Place Reinforcement	Lower surface of cast-in-place concrete	Strain changes in cast-in-place reinforcement during loading, used to evaluate the stress condition of the cast-in-place layer
	Center of the cast-in-place layer	Detection of strain in the center area of the cast-in-place layer to evaluate the overall stress condition
	Edge of the cast-in-place layer	Detection of strain in the edge area of the cast-in-place layer to evaluate the stress condition of the edge area

### Loading device and loading protocol

**Fig. 4:**
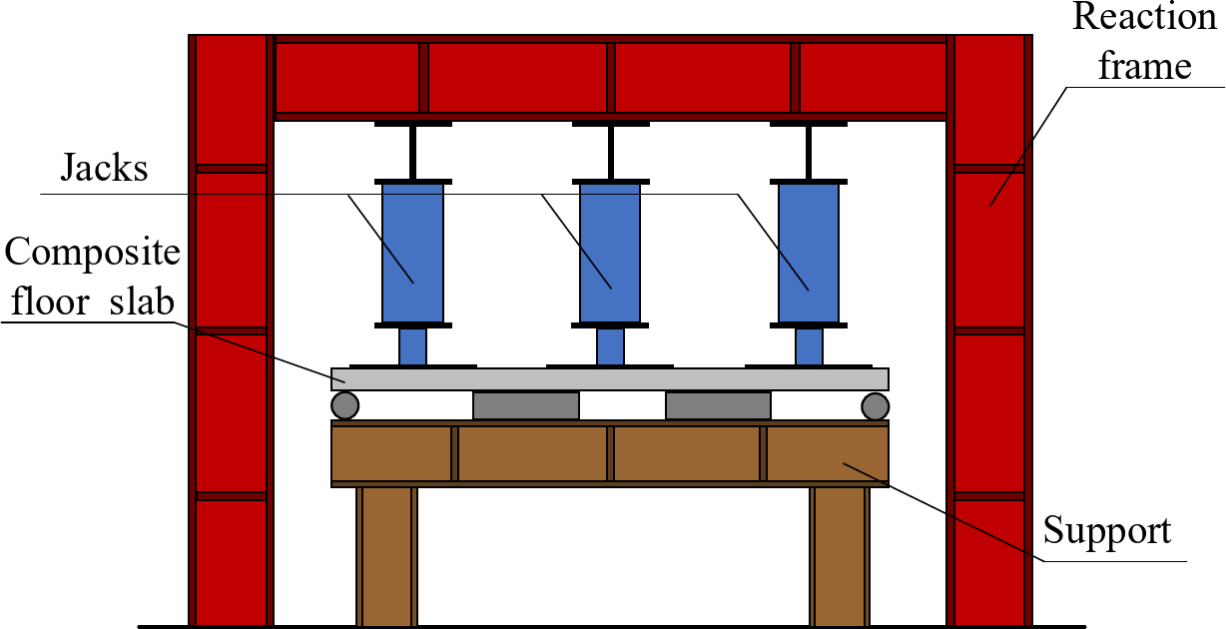
The loading device.

Nine jacks are used for loading in the test. The loading device is shown in Fig. 4. In order to change the concentrated load into uniformly distributed load, a steel plate is set under the jack, and the size of the steel plate is 300 mm × 300 mm × 30 mm, nine pieces in total, which are evenly distributed on the laminated plate as far as possible, and a certain gap (about 50 mm) is reserved between the steel plates to prevent the steel plates from colliding due to the deformation of the test piece. Before the formal loading, the components shall be preloaded to check whether the indication of the instrument is normal. The preloading is carried out in three levels: 2kN / m^2^, 4kN / m^2^ and 6kN / m^2^. After checking that there is no error, unload the test piece in three levels and reset the instrument. Graded loading shall be adopted during formal loading: at level 1–10, the load of each level shall be 2.5% of the calculated value of cracking load and 1.3kN/ m^2^; At level 11–15, the load of each level is changed to 10% of the calculated value of ultimate load, taking 5.2kN/ m^2^; After grade 16, the load of each grade is taken as 4% of the calculated value of ultimate load, taking 2kN / m2 until the test piece breaks the ring.

While the loading protocol was designed to ensure uniform load distribution and accurate measurement, several potential sources of error could still affect the experimental results. Variations in the placement of the steel plates or the underlying slab surface could lead to uneven load distribution. To mitigate this, the steel plates were carefully leveled and aligned before loading to ensure uniform contact with the slab surface^20^. Moreover, friction between the steel plates and the slab surface could affect the loading accuracy. To minimize this, a thin layer of lubricant was applied to the contact surfaces to ensure smooth load transfer. Additionally, the accuracy of the load measurement depends on the calibration and proper functioning of the load cells and other measuring instruments. All instruments were calibrated before the test, and a preloading stage was conducted to verify the normal operation of the instrumentation. Furthermore, the deformation of the slab under load could cause the steel plates to collide, leading to uneven load distribution. To prevent this, a gap of approximately 50 mm was maintained between the steel plates to allow for independent movement during the loading process. Finally, the boundary conditions of the slab, such as the support conditions and the constraints at the edges, could significantly influence the load distribution. To ensure accurate boundary conditions, the slab was carefully supported on all sides, and the supports were designed to closely match the theoretical assumptions used in the analysis.

### Summary of experimental methods

The experimental setup for the composite floor slab was meticulously designed to ensure accurate and reproducible results. The slab specimen measured 3180 mm in length and 3080 mm in width, with a total thickness of 130 mm. This thickness was composed of a 60 mm precast bottom slab and a 70 mm cast-in-situ concrete layer. The precast bottom slabs were spaced 140 mm apart to accommodate the composite structure.

In terms of material specifications, the concrete used for both the precast and cast-in-situ layers was standard structural concrete, although the exact grade was not specified in the original document. The reinforcement included a90 specification trusses for the precast bottom slab, and additional reinforcement mesh sheets were strategically placed at the joints to enhance structural integrity and crack resistance. Specifically, the mesh sheets at Joint 1 were spaced at 97 mm, while those at Joint 2 were spaced at 200 mm.

The loading conditions were carefully controlled to simulate real-world applications. Nine jacks were employed to apply the load, with each jack supported by a 300 mm × 300 mm × 30 mm steel plate to distribute the load uniformly across the slab. To ensure the accuracy of the loading process, a preloading phase was conducted in three stages: 2 kN/m², 4 kN/m², and 6 kN/m². This step verified the normal operation of the instrumentation. After confirming no errors, the specimen was unloaded, and the instruments were reset. The formal loading then proceeded in stages: Levels 1 to 10 applied a load of 2.5% of the calculated cracking load (1.3 kN/m²); Levels 11 to 15 increased the load to 10% of the calculated ultimate load (5.2 kN/m²); and from Level 16 onwards, the load was set at 4% of the ultimate load (2 kN/m²) until the specimen failed.

## Experimental phenomenon

**Fig. 5:**
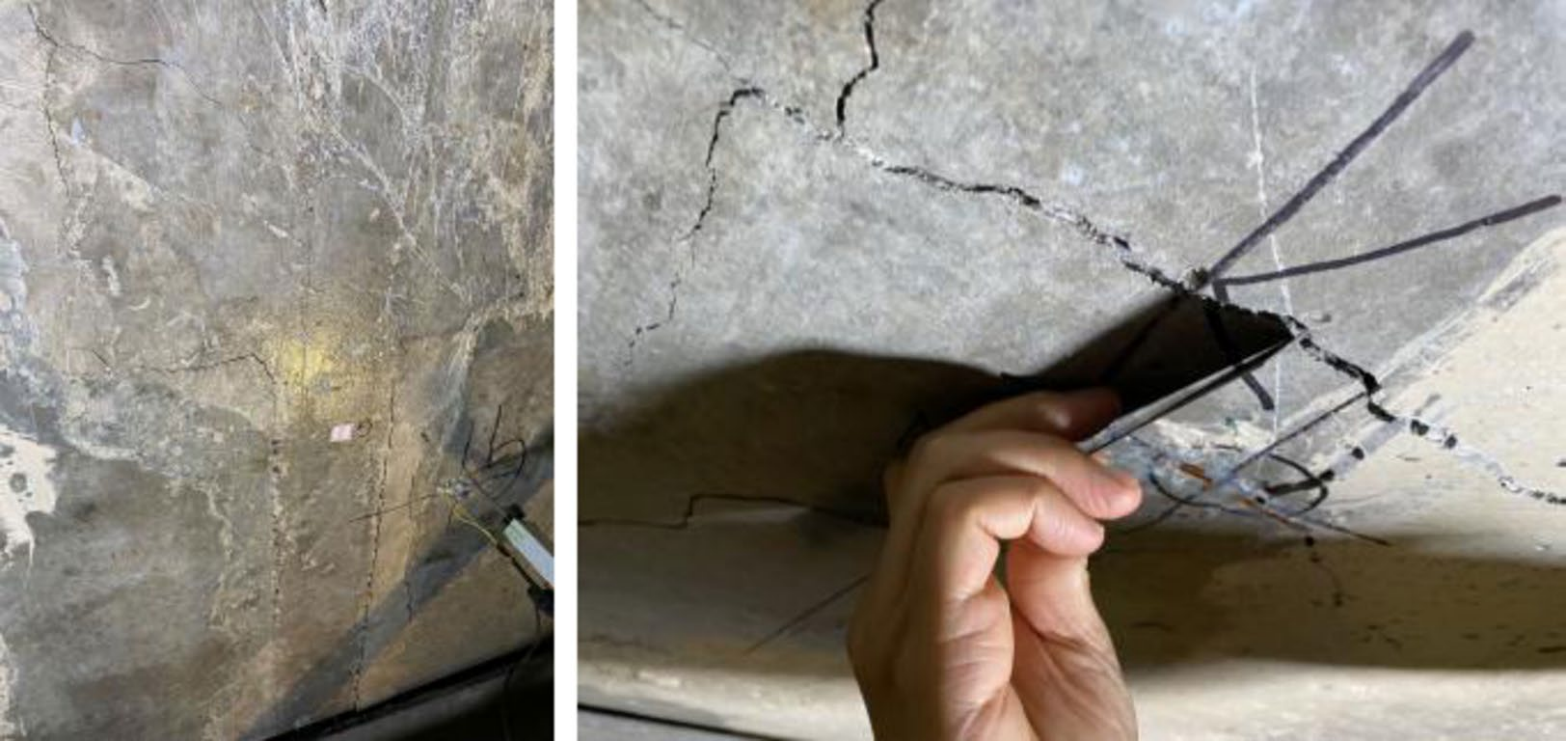
The concrete cracks.

Before the load reaching to 13 kN / m^2^, the specimen is basically in elastic state, the specimen has almost no change, and the load displacement basically increases linearly; When the floor slab load is 1.5 kN / m^2^, 2.0 kN / m2, 2.5 kN / m^2^ and 5.0 kN / m^2^ respectively, the vertical deflection at the corresponding floor slab center point is 0.077 mm, 0.103 mm, 0.128 mm and 0.481 mm respectively. When the floor slab load reaches 18.2 kN / m2, cracks appear at the bottom of the floor slab at the cast-in-situ belt. With the increase of the load to 23.4 kN / m^2^, cracks gradually appear in the diagonal direction of the floor slab, and the stress of the floor slab enters the elastic-plastic stage; When the deflection of the center point of the floor slab reaches 65 mm, and the mid span displacement has exceeded 1 / 50 of the longer side span, it can be considered that the composite floor slab has reached the ultimate bearing capacity state, and the floor slab specimen still maintains a high bearing capacity at this time; When the deflection of the center point of the floor slab reaches 94 mm, and the mid span displacement has exceeded 1 / 50 of the longer side span, it can be considered that the composite floor slab has reached the peak load, and the corresponding ultimate bearing capacity is 51kN / m^2^. At this time, there has been serious cracking at the bottom of the floor slab, which can be considered to have reached the ultimate failure state. Figure 5 shows the concrete cracks.

The crack patterns observed in the new composite floor slab with split joints provide valuable insights into its structural behavior, particularly when compared to standard composite slabs. In traditional composite slabs, cracks generally initiate at the bottom of the slab under similar loading conditions and tend to propagate in a more random and less controlled manner. This is often due to the continuous reinforcement layout, which can lead to higher stress concentrations at certain points.

**Fig. 6:**
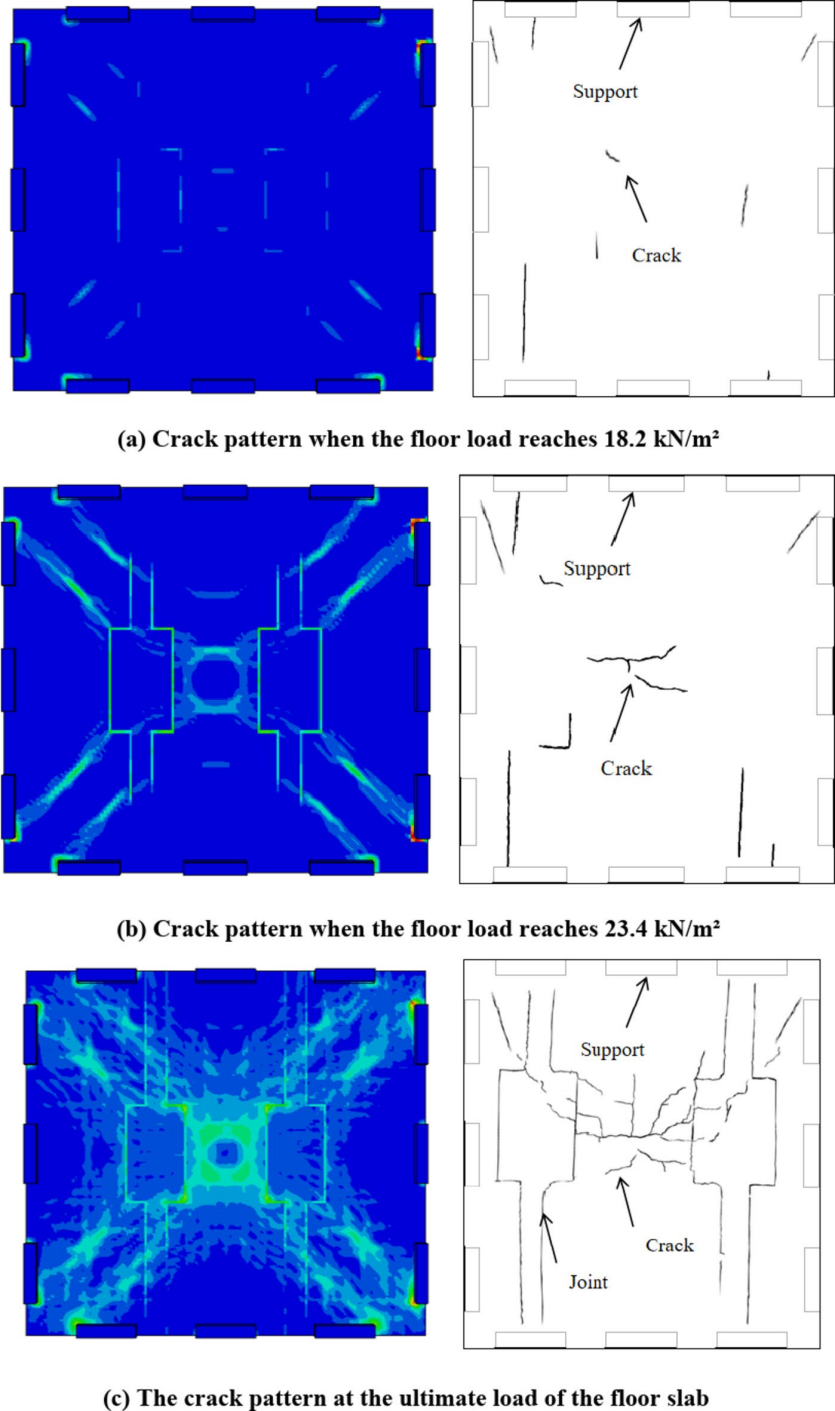
Top view of crack development in the floor slab at different loading stages.

In contrast, the crack patterns in the new composite floor slab with split joints show a more controlled and predictable distribution. Cracks first appeared in the cast-in-situ belt under a load of 18.2 kN/m² (as shown in Fig. 6(a))and then extended diagonally as the load increased(as shown in Fig. 6(b)). This pattern suggests that the centralized reinforcement layout at the joints effectively controls the initiation and propagation of cracks. The split joint design, with its optimized reinforcement distribution, appears to mitigate the risk of uncontrolled crack growth, thereby enhancing the overall structural integrity and durability of the slab.Furthermore, the new composite floor slab achieved an ultimate failure load of 51 kN/m², which is higher than that of typical standard composite slabs(as shown in Fig. 6(c)). The crack propagation stage is shown in Fig. 6.The latter often fail at lower load levels due to more extensive cracking and a loss of load-carrying capacity. This indicates that the new design not only controls crack patterns but also significantly improves the load-carrying capacity and safety reserve of the composite slab.In summary, the crack patterns observed in the new composite floor slab with split joints demonstrate superior performance compared to standard composite slabs. The centralized reinforcement layout and split joint design effectively control crack initiation and propagation, resulting in enhanced structural integrity, higher load-carrying capacity, and improved durability. These findings underscore the benefits of the new design in practical engineering applications, especially in scenarios where crack control and structural performance are critical.

## Analysis of test results

### Uniform load vertical Deflection curve

The loading process of composite floor slab can be divided into three stages: elastic stage, yield stage and failure stage. In the elastic stage, the curve develops linearly, the reinforcement is in the elastic state at this stage, and the concrete has cracks at this stage. In the yield stage, the stiffness of the composite floor slab decreases obviously, and the growth rate of vertical displacement accelerates; In the failure stage, the load displacement curve becomes more gentle and the deflection develops faster until the specimen is damaged.

**Fig. 7:**
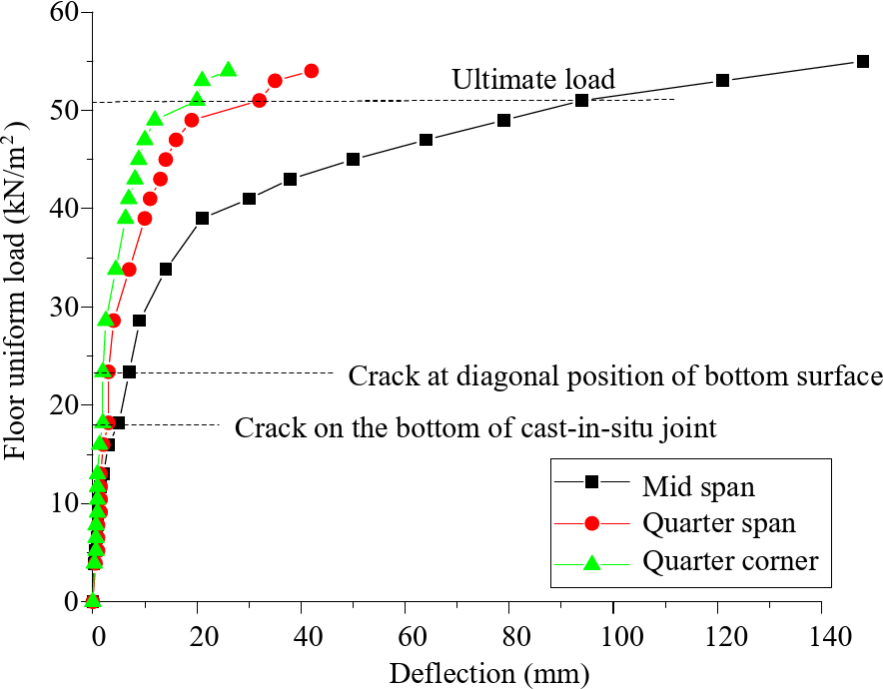
The vertical deflection of floor slab under uniform load.

In the test, in addition to the center of the composite slab, the vertical deflection displacement measuring points are arranged at the 1 / 4 span and the 1 / 4 span near the corner, and the floor slab load vertical deflection curves at different positions are obtained, as shown in Fig. 7. Under the same load, the deflection of the diagonal position is less than that of the 1 / 4 span position in the long span direction, and the vertical deflection of the 1 / 4 span position in the long span direction is about 1 / 5 of that in the middle of the span. After reaching the ultimate bearing capacity, the continuously loaded floor slab still has large stiffness, and the bearing capacity does not decrease significantly, indicating that the composite floor slab has high safety reserve.

To further assess the practical relevance of the experimental results, the measured deflections were compared with the standard deflection limits specified in engineering design codes. According to the relevant provisions of the Chinese National Standard GB 50,010 − 2010 Code for Design of Concrete Structures (Ministry of Housing and Urban-Rural Development of China, 2010), which is widely used for the design and assessment of reinforced concrete structures in China, the allowable deflection limit for composite floor slabs under service loads is typically expressed as a fraction of the span length. For example, the deflection limit for a simply supported slab is often specified as L/250 or L/300 of the longer span, where L is the span length of the slab. These limits are comparable to those recommended in other international codes such as Eurocode 2 (EN 1992) and ACI 318, which also suggest similar ratios for reinforced concrete slabs.

In this study, the maximum measured deflection at the center of the slab under the ultimate load was 65 mm. Given the span length of the composite floor slab (3180 mm in the longer direction), the deflection ratio is approximately L/49. This value is well within the allowable limits specified in the design codes, indicating that the new type of composite floor slab with split joints not only exhibits good structural performance but also meets the practical requirements for deflection control in engineering applications. The use of GB 50,010 − 2010 in this study provides a conservative benchmark for assessing the structural performance of composite floor slabs and ensures the practical relevance of the findings.

### Analysis of concrete strain curve

**Fig. 8:**
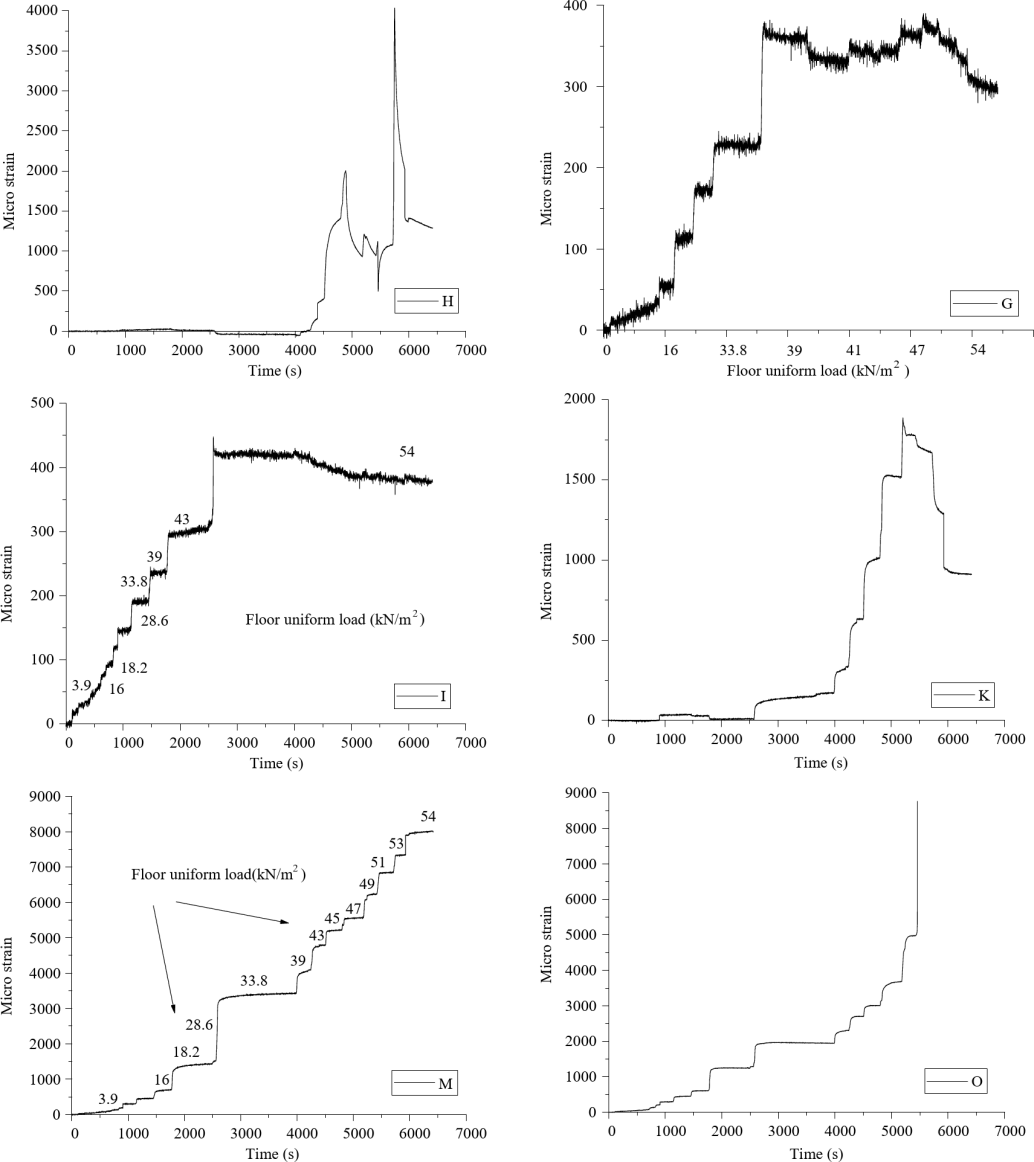
The concrete-strain curve under floor uniform load.

Data are collected from the bottom concrete strain measuring points and the curve between slab uniformly distributed load and concrete strain is shown in Fig. 8. Under the uniformly distributed load on the slab, the concrete strain on both sides of the joint is basically coordinated, which proves that the joint connection form can better transfer the stress between the two precast slabs. The laminated slab has good integrity and the stress form is similar to that of two-way slab. In Fig. 3(a), measuring points h and K are located at the cast-in-situ joint, so when the uniformly distributed load of the slab reaches 47kN / m^2^, there is a crack passing through the concrete strain gauge, resulting in the damage of the concrete strain gauge, and the micro strain of the concrete decreases in the later stage. Measuring points G, I, M and O are located near the diagonal of the slab. When diagonal cracks appear in the strain gauge during loading, the data of this measuring point exceeds the limit or decreases. Comparing the concrete strain values of each measuring point under the same load, it seems that the slab has typical stress and failure characteristics of two-way slab.

To further validate the findings, a detailed comparison between the experimental and finite element method (FEM) results was conducted. For example, at a load level of 18.2 kN/m², the experimental strain values at measuring points h and K were 25.6 µε and 28.4 µε, respectively. The corresponding FEM-predicted values were 24.8 µε and 27.9 µε, with percentage differences of 3.1% and 1.8%, respectively. Similarly, at a higher load level of 47 kN/m², the experimental strain values at points G and I were 78.3 µε and 82.1 µε, while the FEM-predicted values were 77.2 µε and 81.3 µε, resulting in differences of 1.4% and 1.0%. These minor discrepancies can be attributed to the idealized assumptions in the FEM model, such as material homogeneity and boundary conditions. Overall, the close agreement between the experimental and FEM results confirms the reliability of the numerical model and strengthens the findings regarding the mechanical behavior of the composite slab.

### Analysis of load-strain curve of prefabricated bottom slab reinforcement

The connecting reinforcement at the joint of the superimposed slab is the main connecting force transfer reinforcement between the prefabricated bottom plates. This paper analyzes its strain during the test (see Section II for the specific number). After the test, the uniformly distributed load strain curve of the connecting reinforcement is obtained by processing the data, as shown in Fig. 12. The stress of the connecting reinforcement at the joint 2 in the superimposed slab changes greatly, so it can be seen that the connecting reinforcement is an important force transfer structure between the two precast slabs; In addition, with the different distance between the connecting reinforcement and the center of the slab, the strain of the reinforcement under the same load is also different. The closer the connecting reinforcement to the center of the slab, the greater the strain and the earlier the yield occurs; Similarly, the strain of connecting reinforcement far away is small. For example, at measuring point 24, the yield strain can be reached only when the uniformly distributed load on the slab reaches 51kN / m^2^.

**Fig. 9:**
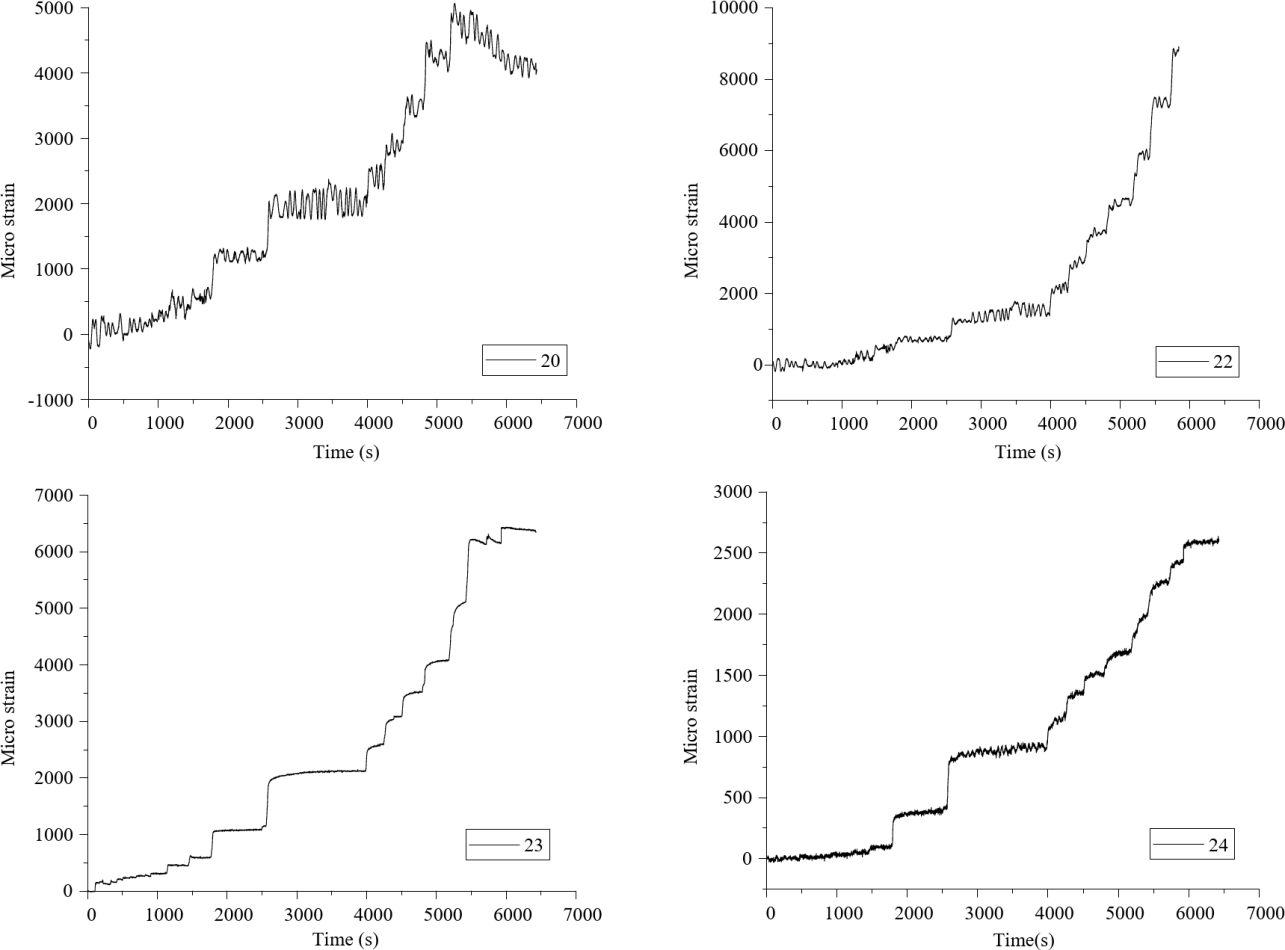
Load-strain curve of prefabricated bottom slab reinforcement.

The analysis of the load-strain curves indicates that the connecting reinforcement’s performance is highly dependent on its location relative to the slab center and joint details. Since reinforcement closer to the slab center experiences higher strain and earlier yielding, the current layout may not be optimal for uniform load distribution. To enhance structural efficiency, several strategies could be explored. Adjusting the reinforcement layout to increase density near the slab center while maintaining adequate spacing elsewhere could reduce stress concentration and improve load-carrying capacity. Additionally, using high-strength reinforcement could delay plastic deformation and enhance performance. Staggering reinforcement bars along the joint could also reduce stress concentration and improve load transfer between precast slabs. Optimizing joint geometry or adding shear reinforcement could further enhance load transfer and reduce strain concentration. Finally, an adaptive reinforcement design informed by finite element analysis could lead to more efficient material usage and better structural performance. In summary, optimizing reinforcement detailing based on load-strain behavior could significantly improve load distribution and overall structural efficiency. Future work should focus on implementing and testing these strategies to validate their effectiveness in enhancing composite floor slab performance.

### Analysis of load-strain curve of reinforcement in laminated concrete

**Fig. 10:**
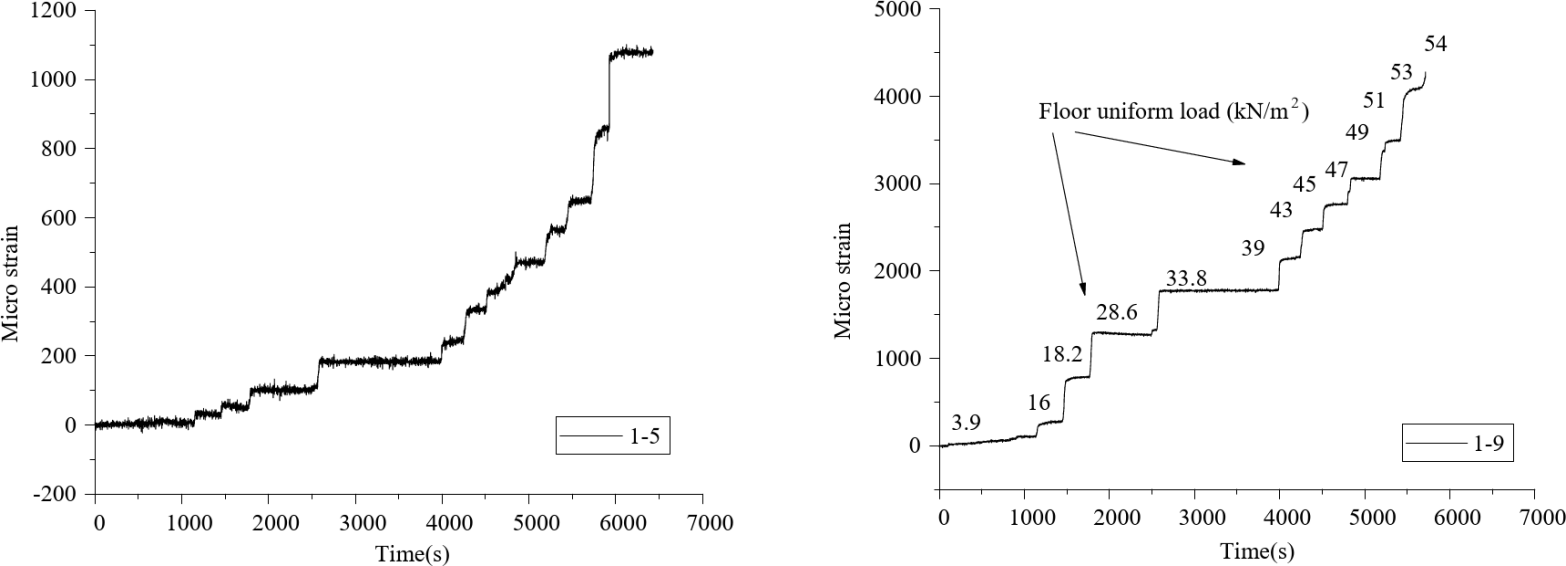
Load-strain curve of reinforcement at joint.

This new type of composite slab is provided with reinforcement mesh at the joint for crack prevention and auxiliary force transmission. In the test, the paving reinforcement strain at joint 1 and joint 2 is also measured (Figs. 2 and 3). After the test, the uniformly distributed load reinforcement strain curve of the composite slab is obtained through data processing. In Figs. 1, 2, 3, 4, 5, 6, 7, 8, 9 and 10 are the steel bar strain measuring points in the joint 2, and 1–5 are the steel bar strain measuring points at the joint 1. It can be seen from the Fig. that under the same slab load, the strain value of measuring points 1–9 is greater. When the uniformly distributed load of the slab reaches 39kN / m^2^, the tensile strain of the reinforcement at measuring points 1–9 has reached yield, the reinforcement at measuring points 1–5 has not yielded, and the reinforcement at measuring points 1–5 has not yielded at ultimate load. This shows that the crack prevention reinforcement laid at the joint has formed a crack prevention mechanism, which can effectively slow down the development of cracks at the bottom of the slab. The closer it is to the center of the slab, the greater the tensile strain of the paved reinforcement, and the more obvious the crack prevention effect.

The observed strain distribution and crack development in the composite slab suggest that further optimization of the reinforcement layout could enhance crack prevention. For instance, increasing the density of reinforcement mesh at the joint, particularly in areas closer to the slab center where higher strains are observed, could provide additional resistance to crack propagation. Additionally, using reinforcement with higher tensile strength or incorporating fiber-reinforced concrete in the joint area could further improve the slab’s crack resistance. Another potential approach is to introduce a multi-layered reinforcement configuration, where additional reinforcement layers are placed at different depths within the joint to create a more robust crack prevention mechanism. This could help distribute the tensile forces more evenly and reduce the likelihood of crack formation. Overall, exploring alternative reinforcement layouts and materials could significantly enhance the crack prevention capabilities of the composite slab, contributing to its improved durability and serviceability.

## Finite element numerical simulation modeling

**Fig. 11:**
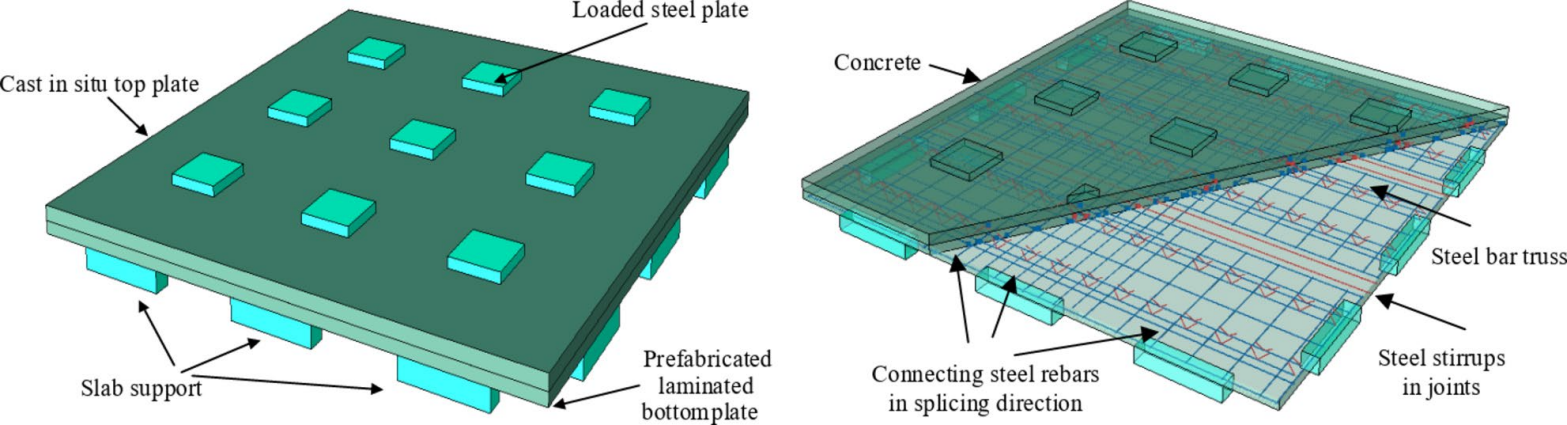
Finite element model diagram.

ABAQUS (Advanced Simulation for Engineering and Sciences Unified Finite Element Analysis Solution, ABAQUS 6.14-4,https://www.3ds.com/products/simulia/abaqus)finite element software is used to.

establish the finite element model of the new type of jointed composite slab. The prefabricated concrete slab, laminated concrete, loaded steel plate and slab support are simulated by eight node linear reduced integral hexahedral solid element (C3D8R), and the reinforcement and truss in the slab are simulated by three-dimensional truss element (T3D2). Finally, a total of 567,890 elements are set in this model, As shown in Fig. 11.

To ensure the accuracy and reliability of the finite element analysis, a convergence study on mesh density was conducted. The study involved incrementally refining the mesh density in critical areas, such as the joints and regions with high stress gradients, and evaluating the impact on the results. It was found that further refinement of the mesh beyond a certain density did not significantly alter the results, indicating that the chosen mesh density was sufficient to capture the structural behavior accurately. The final mesh density was selected based on this study to balance computational efficiency and accuracy.

In the material properties used for the finite element model, certain assumptions were made to simplify the analysis. For instance, the effects of concrete shrinkage and creep were ignored. This simplification was based on the assumption that these long-term effects are relatively minor compared to the immediate structural response under the applied loads. However, it should be noted that in real-world applications, shrinkage and creep can have significant impacts on the long-term performance of concrete structures, particularly in terms of crack formation and deflection. Therefore, while the current model provides valuable insights into the structural behavior under short-term loading, future work should consider incorporating these effects to enhance the model’s applicability to long-term structural performance.

Plastic damage model is adopted for concrete, and relevant parameters are shown in Table 2. The constitutive relationship of concrete adopts the model recommended in GB 50,010 − 2010 code for design of concrete structures^21^, and the constitutive relationship of reinforcement adopts the ideal elastic-plastic model.

**Table 2:** Parameters of concrete plastic damage model.

dilation angle	Eccentricity	f_b0_/f_c0_	k	Viscosity coefficient
30	0.1	1.16	0.6667	0.0005

Face to face binding constraint (TIE) is adopted between the precast bottom plate and the laminated concrete, and the loaded steel plate is also bound with the laminated concrete by tie constraint, while the reinforcement is embedded in the concrete. The section normal direction of the slab support and the precast bottom plate is set as “hard” contact, the tangential direction is set as friction contact, and the friction coefficient is 0.6^22^; The boundary condition is to constrain all nodes at the bottom of the slab support, and the loading method is to apply concentrated force at the center of the loaded steel plate.

## Analysis of finite element result analysis

### Load-deflection curve analysis

**Fig. 12:**
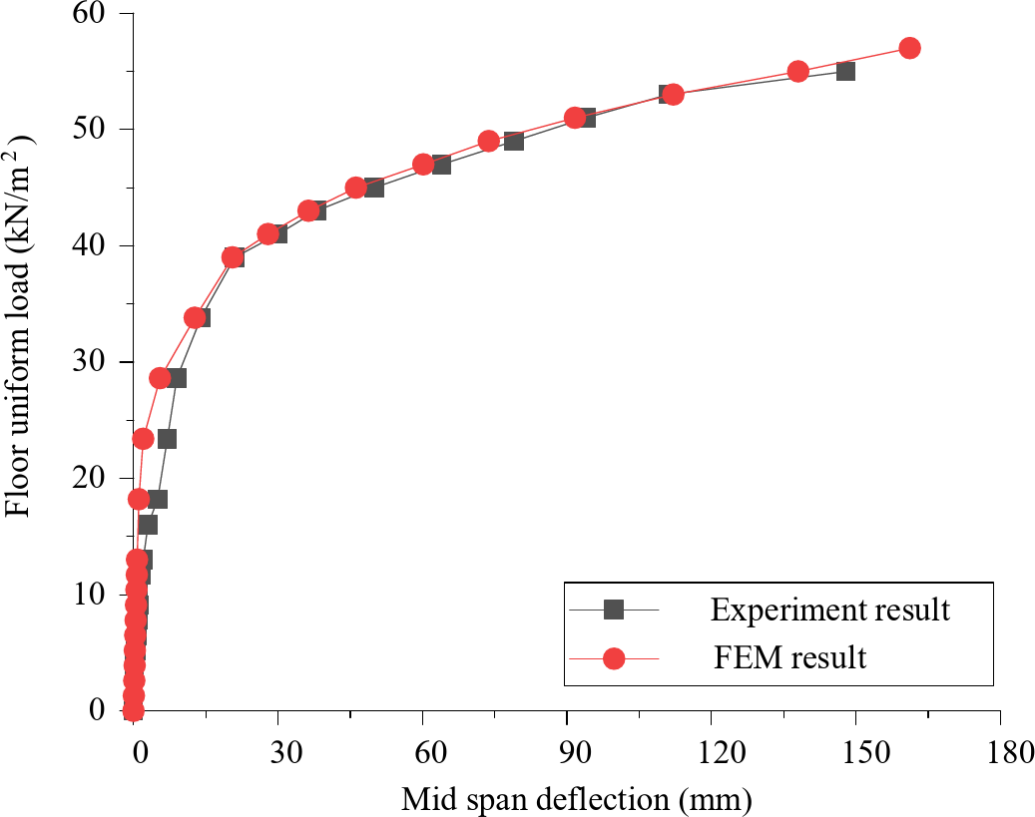
Load-deflection curve comparison.

In order to verify the accuracy of the finite element numerical simulation, the variation curve of the vertical deflection of the center point at the bottom of the slab with the slab load is extracted and compared with the test results. The results show that the finite element simulation results are in good agreement with the test, as shown in Fig. 12. The finite element numerical simulation can provide a basis for subsequent research and analysis.

To further enhance the robustness of the finite element model, a sensitivity analysis was performed on key parameters, including contact stiffness and boundary conditions. The analysis focused on the contact stiffness between the precast bottom plate and the laminated concrete, as well as between the loaded steel plate and the slab. Variations in contact stiffness were found to have a significant impact on the predicted load-deflection behavior. Specifically, increasing the contact stiffness led to a stiffer response, with reduced deflection values, while decreasing the stiffness resulted in a more flexible behavior and higher deflections. Additionally, minor adjustments to the boundary conditions, such as changes in the support constraints or friction coefficients, also influenced the load-deflection curves. For example, increasing the friction coefficient from 0.6 to 0.8 resulted in a noticeable reduction in the predicted maximum deflection. These findings highlight the importance of accurately defining these parameters to ensure the reliability of the simulation results. Future work should include further refinement of these parameters based on additional experimental data and sensitivity analyses to improve the model’s accuracy and robustness.

### Stress-strain analysis of concrete at the bottom of slab

**Fig. 13:**
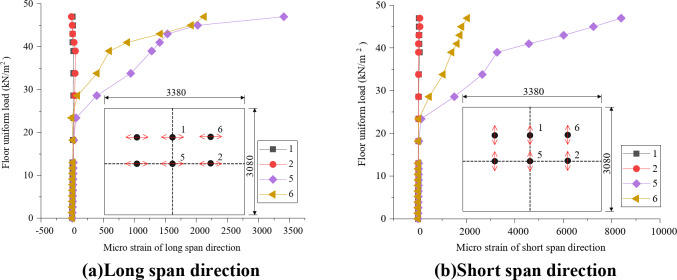
Strain curve of bottom slab.

Figure 13 shows that the concrete strain at the center of the slab is the largest, followed by the diagonal position of the slab, and the concrete strain at one quarter of the long span and short span is at a low level. It can be concluded that: firstly, the composite slab has good integrity, and the joint structure can carry out good stress transfer; Secondly, the stress and deformation of the composite slab under the uniformly distributed load of the slab show the characteristics of two-way slab. The center of the slab is the middle of the span of short span and long span, so the strain and stress are the largest; The strain of measuring point 6 is also at a large level, because it is located near the plastic hinge line of two-way slab. With the increase of load, the diagonal position will crack.

The stress-strain analysis presented above focuses on the immediate structural response of the composite slab under short-term loading conditions. However, it is important to consider the potential long-term effects that could influence the stress distribution within the slab. For instance, creep and shrinkage, which are common time-dependent behaviors of concrete, may significantly alter the stress state over time. Creep, characterized by the gradual deformation of concrete under sustained load, could lead to a redistribution of stresses, particularly in regions with high initial stress concentrations, such as the slab center and diagonals. Similarly, shrinkage, which is the tendency of concrete to contract over time, could induce additional tensile stresses, potentially exacerbating crack formation and propagation. These long-term effects could reduce the overall stiffness of the slab and affect its load-carrying capacity. Therefore, while the current analysis provides valuable insights into the short-term behavior, future work should incorporate long-term material behaviors such as creep and shrinkage to offer a more comprehensive understanding of the composite slab’s performance over its service life.

### Stress-strain analysis of reinforcement in composite slab

**Fig. 14:**
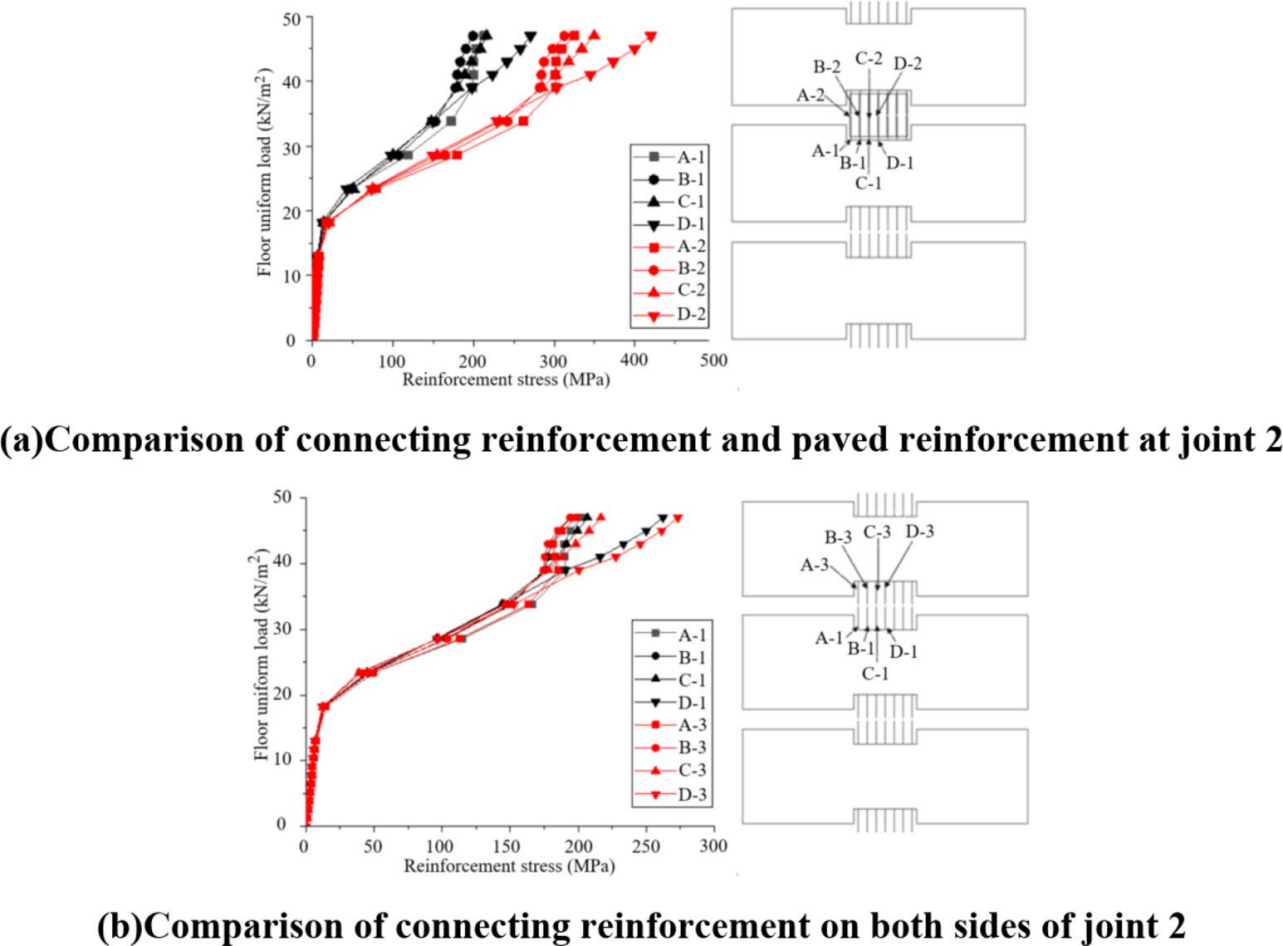
Load stress curve of reinforcement.

In order to analyze the force transmission mechanism and force change law of the reinforcement at the joint in the composite slab, the stress change curve of the connecting reinforcement and the paving reinforcement at the joint 2 is extracted for analysis, as shown in Fig. 13. Figure 14 (a) shows the stress of connecting reinforcement at the joint 2 and the adjacent upper layer paving reinforcement. It can be seen that the tensile stress of the connecting reinforcement is greater than the paving reinforcement under the uniformly distributed load, indicating that the connecting reinforcement is the main force transfer structure at the joint of the superimposed slab; Fig. 14 (b) shows the stress comparison curve of connecting reinforcement on both sides of joint 2. It can be seen from the curve that the stress of connecting reinforcement on both sides of joint 2 at the same position is relatively close, indicating that the joint structure in this study can better ensure the force transmission between precast bottom plates. In addition, the closer the curve in the Fig. is to the center of the plate, the greater the stress of connecting reinforcement. This phenomenon is consistent with the experimental observation, It also shows that the composite slab structure proposed in this study has the mechanical characteristics of two -way slab.

The analysis of the stress-strain behavior of the reinforcement highlights the importance of reinforcement spacing in influencing the overall performance of the composite slab. In the current design, the connecting reinforcement and paving reinforcement play crucial roles in load transfer and crack prevention. The spacing of these reinforcement bars directly affects the stress distribution and, consequently, the structural integrity and durability of the slab. Closer reinforcement spacing, particularly in high-stress regions such as the slab center and joints, can lead to more uniform stress distribution and reduced risk of crack formation. This is because closely spaced reinforcement bars can better resist tensile forces and limit crack width. Conversely, wider spacing may result in higher local stresses and increased likelihood of cracking, especially under long-term loading conditions. Therefore, optimizing reinforcement spacing based on stress analysis and experimental observations can significantly enhance the load-carrying capacity and crack resistance of the composite slab. Future work should focus on conducting parametric studies to determine the optimal reinforcement spacing for different regions of the slab, considering factors such as load magnitude, slab geometry, and material properties. This will provide valuable insights for practical engineering applications and contribute to the development of more efficient and durable composite slab designs.

**Fig. 15:**
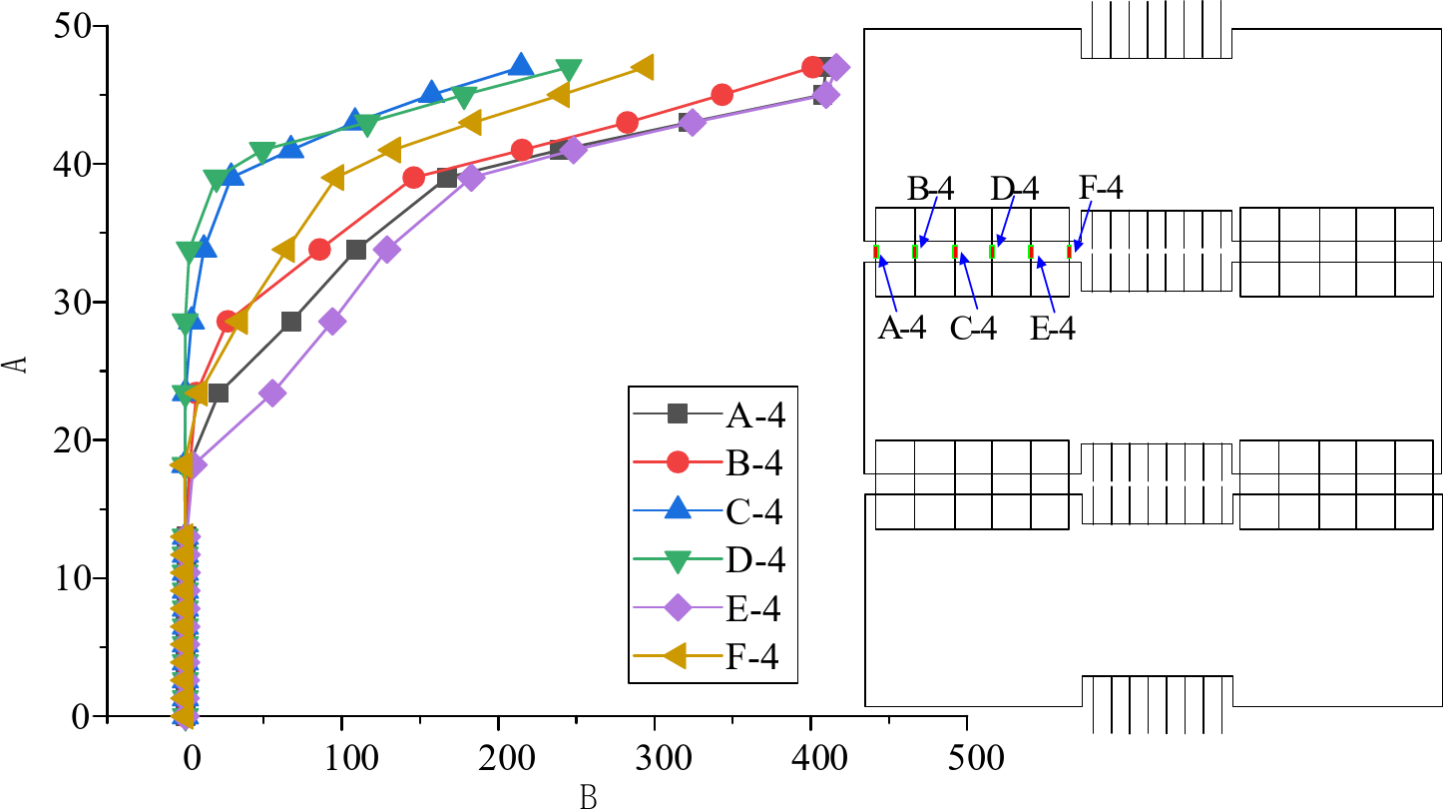
The load-stress curve of paved reinforcement at joint 1.

In addition, according to the finite element model, the reinforcement stress outside the joint 2 position is analyzed. Figure 15 shows the strain change of the paved reinforcement above the joint 1 along the long span direction of the slab. It can be seen that the stress of the reinforcement closer to the central point of the slab is greater, and the stress value has been close to the yield strength of the reinforcement, indicating that the paved reinforcement also has a large force transfer function in this joint structure.

### Cloud image analysis

#### Concrete damage nephogram

**Fig. 16:**
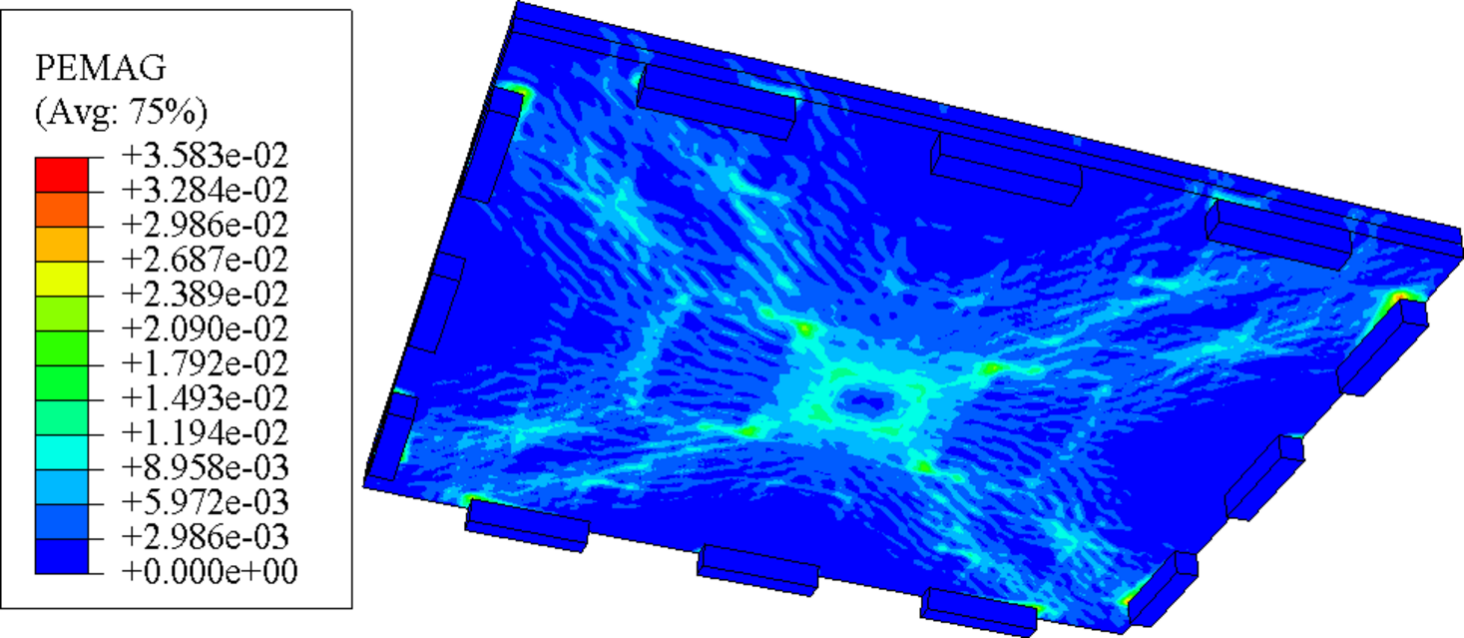
The damage nephogram of the concrete.

The damage nephogram of the concrete at the bottom of the slab can show the development of cracks. It can be seen from Fig. 16 that the new type of jointed composite slab has significant crack distribution and failure characteristics of two-way slab: when the uniformly distributed load of the slab is 18.2kN/m^2^, the concrete at the bottom of the slab center cracks, and the plastic strain also occurs at the diagonal(as shown in Fig. 6(a)). With the increase of the load, the plastic area at the slab center and diagonal increases and expands until the slab reaches the ultimate bearing capacity(as shown in Fig. 6); The equivalent plastic strain at the four corners is the largest, and there is a large equivalent plastic strain along the edge of the precast base plate, indicating that there is a sudden change in the stiffness of the concrete at the precast base plate and the post cast strip, and the cracks of the composite slab should be mainly concentrated at the joint between the plastic hinge line and the base plate.

To validate the conclusions drawn from the finite element analysis, the simulated crack patterns were compared with the experimental crack patterns observed during the loading tests. The experimental results showed that cracks initially appeared at the bottom center of the slab under a load of 18.2 kN/m², consistent with the finite element simulations. As the load increased, the cracks propagated diagonally, aligning well with the simulated plastic strain distribution. The close agreement between the simulated and experimental crack patterns confirms the accuracy of the finite element model in predicting the failure mechanisms of the composite slab. Minor discrepancies were observed in the width and distribution of the cracks, which can be attributed to the idealized assumptions in the finite element model, such as material homogeneity and boundary conditions. Overall, the comparison strengthens the conclusions regarding the crack development and structural behavior of the composite slab.

#### Yield nephogram of reinforcement mesh

**Fig. 17:**
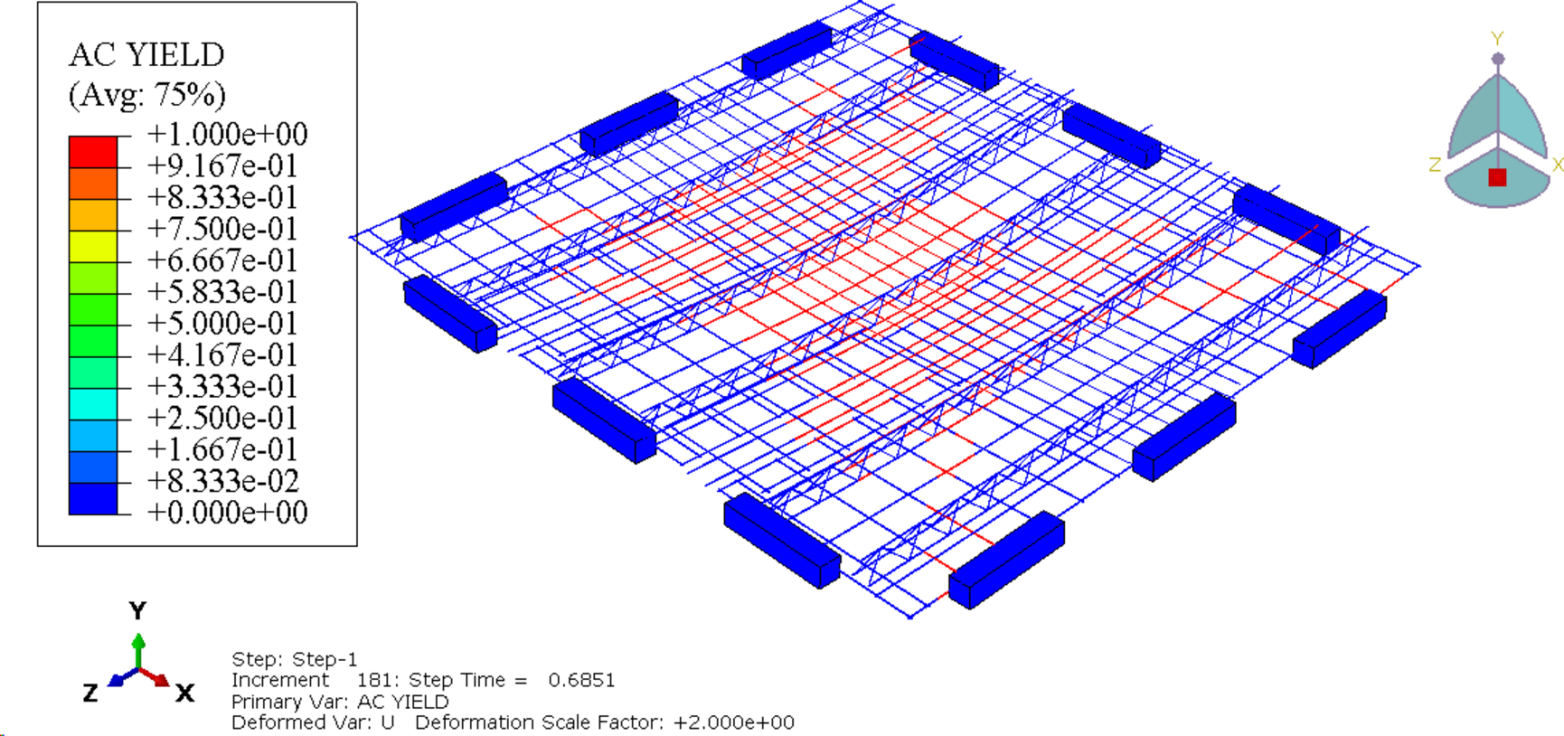
The stress nephogram of the reinforcement mesh.

According to the stress nephogram of the reinforcement mesh at the bottom of the slab (Fig. 17), the tensile stress of the reinforcement mesh is mainly distributed along the plastic hinge line of the slab, and the maximum tensile stress has reached the set yield limit, which corresponds to the yield of the tensile reinforcement in the crack zone in the plastic hinge line theory. The lower chord of the steel truss bears tensile stress, the upper chord bears compressive stress, while the web reinforcement bears relatively small stress, which is between tension and compression, and only the lower chord of the steel truss has large plastic deformation, indicating that the deformation of the laminated plate mainly occurs in the prefabricated bottom plate.

#### Concrete damage nephogram

**Fig. 18:**
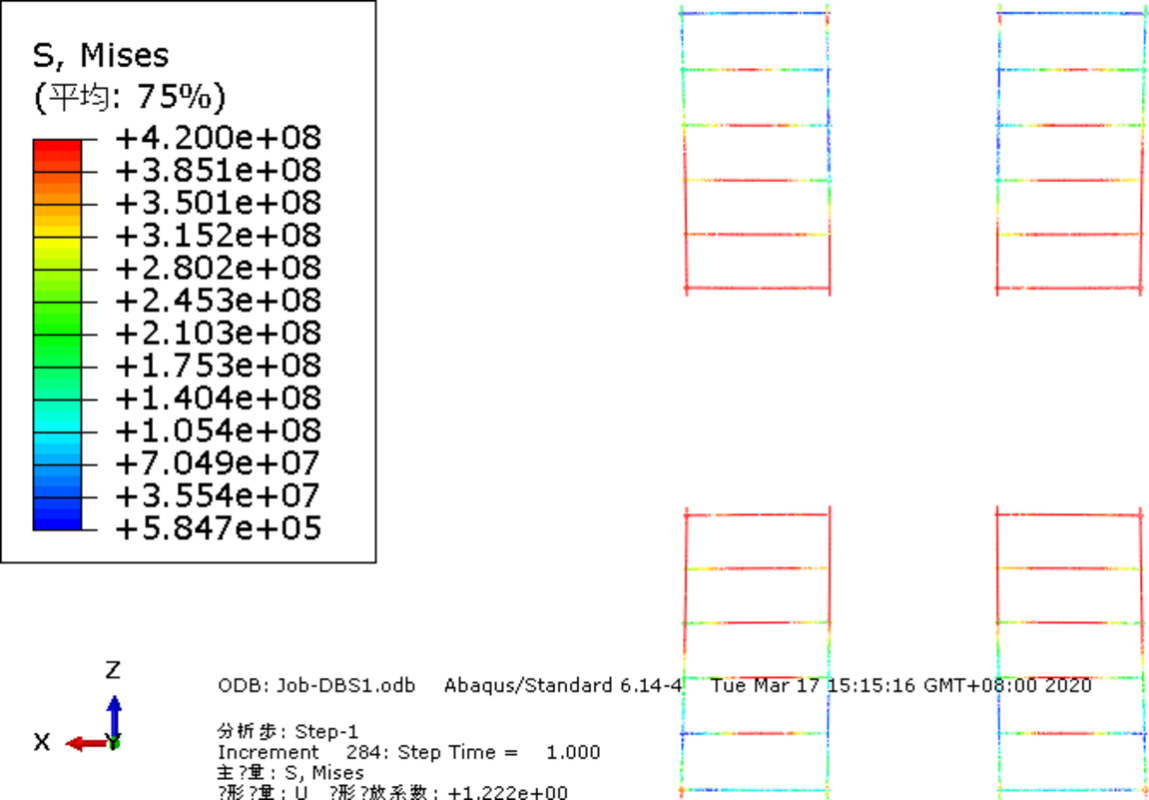
The stress nephogram of the paved reinforcement mesh(Generated using ABAQUS 6.14-4).

According to the stress nephogram of the paved reinforcement mesh and the damage nephogram of the anti crack reinforcement mesh (Fig. 18), the paved reinforcement mesh has reached the yield limit and produced large plastic deformation. Compared with the concrete at the post cast strip, the paved reinforcement mesh bears greater tensile stress, which indicates that a crack prevention mechanism has been formed. The crack prevention mechanism produced by the paved reinforcement mesh changes the stress characteristics at the joint of the laminated plate, so that the crack at the bottom of the plate will tend to develop along the horizontal direction of the laminated surface when it develops to the laminated surface, and slow down the development of the crack in the vertical direction to prevent the formation of through cracks.

### Comparative analysis of reinforcement and concrete damage behaviour

In this section, the damage behaviour of reinforcement and concrete obtained from experimental and analytical tests is compared to validate the accuracy of the finite element model and to provide a comprehensive understanding of the mechanical performance of the new composite floor slab.

#### Concrete damage behaviour

The concrete strain and crack patterns observed in the experimental tests are compared with those from the finite element simulation. In the experimental tests, cracks were first observed at the bottom of the slab at a load of 18.2 kN/m², with the plastic strain also occurring at the diagonal positions. As the load increased, the crack patterns expanded, particularly at the slab center and diagonals. The finite element simulation results show a similar trend in crack development, with the damage nephogram indicating significant plastic strain at the slab center and diagonals (see Fig. 16). However, minor differences were observed in the crack width and distribution, which can be attributed to the idealized assumptions in the finite element model.

#### Reinforcement stress and strain behaviour

The stress and strain behaviour of the reinforcement in the experimental tests and finite element simulation are also compared. The load-strain curves of the connecting reinforcement and paved reinforcement at the joints, as shown in Figs. 9 and 10, are analyzed. The experimental results indicate that the connecting reinforcement at the joint 2 experiences higher tensile stress compared to the paved reinforcement, which is consistent with the finite element results. However, the experimental data show a slightly higher strain value at the yield point, which may be due to material variability and boundary conditions in the actual test setup.

#### Summary of comparative analysis

Overall, the experimental and analytical tests show good agreement in terms of concrete damage and reinforcement stress behaviour. The finite element model accurately captures the crack development and stress distribution, validating its reliability for further analysis. However, minor discrepancies in crack width and reinforcement strain highlight the need for further refinement of the model assumptions and material properties.

## Theoretical calculation and analysis

### Cracking load calculation

When calculating the cracking load -way composite slab, the concrete strength grade, the ratio of long and short span of composite slab, the layout of reinforcement and other factors should be comprehensively considered^23^. According to the code, the MCRI calculation formula of cracking moment of reinforced concrete flexural members is^21^:

1 $${M_{cri}}=\gamma {f_{tk}}{W_0}$$

2 $$\gamma =\left( {{\text{0}}{\text{0.7}}+120/h} \right){\gamma _m}$$

3 $${W_0}={I_0}/{y_0}$$

Where is *γ* Is the plastic influence coefficient of the section resistance moment of the concrete member, *γ*_*m*_ is the basic value of plastic influence coefficient of section resistance moment of concrete member, and 1.55 is taken for rectangular section; *f*_*tk*_ is the standard value of concrete tensile strength; *W*_*0*_ is the section resistance moment of the tensile edge of the converted section calculated according to the actual size, *y*_*0*_ is the distance from the gravity center axis of the section to the tensile edge, and *I*_*0*_ is the section centroid moment of inertia.

**Fig. 19:**
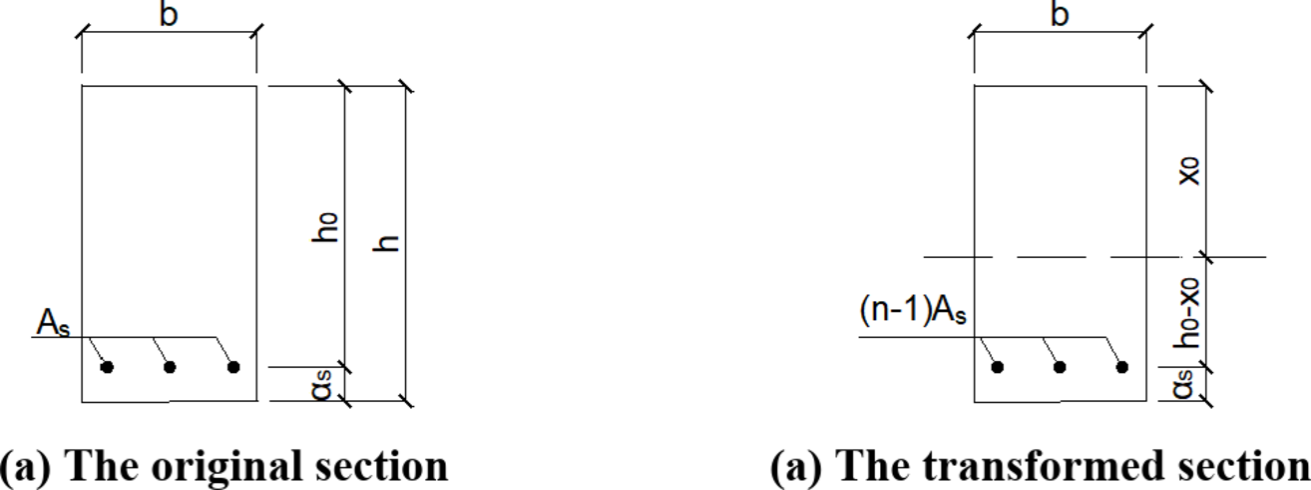
The transformed section before cracking of composite floor slab.

The thickness of the test slab is 130 mm. When h < 400 mm, H = 400 mm^24^. The calculation method of homogeneous elastic material shall be adopted in the design of reinforced concrete members. Its main principle is to convert the cross-sectional area of reinforcement into the cross-sectional area of concrete through the ratio of elastic modulus of reinforcement to concrete^25^. Before the concrete cracks, the full section of the concrete is stressed. The area of reinforcement in the tensile area is as, which is converted into the area of concrete as, where n = ES / EC (ratio of elastic modulus of reinforcement to concrete). When the cross-sectional area of the reinforcement in the tensile area is equivalent to the concrete area, the area of the converted reinforcement itself shall also be subtracted, that is, the actual area to be increased in the concrete tensile area is *(n-1)A*_*s*_.( Fig. 19).

The height x0 of the compression zone is obtained from the area moment conditions of the tension and compression zone to the neutral axis:

4 $$\frac{{\text{1}}}{{\text{2}}}bx_{0}^{2}=\frac{{\text{1}}}{{\text{2}}}b{(h - {x_0})^2}+(n - 1){A_s}({h_0} - {x_0})$$

The moment of inertia of the converted section is:

5 $${I_0}=\frac{b}{{12}}\left[ {x_{0}^{3}+{{(h - {x_0})}^3}} \right]+(n - 1){A_s}{\left( {{h_0} - {x_0}} \right)^2}$$

Since the stress of composite slab is different from that of cast-in-place slab, MCR needs to be adjusted after calculating the cracking moment^26^:

6 $$\left\{ \begin{gathered} {M_{crx}}={{M^{\prime}}_{crx}}+0.2{{M^{\prime}}_{cry}} \hfill \\ {M_{cry}}={{M^{\prime}}_{cry}}+0.2{{M^{\prime}}_{crx}} \hfill \\ \end{gathered} \right.$$

The smaller value of cracking load in two directions is taken, and the calculation formula is:

7 $$\left\{ \begin{gathered} {q_{crx}}={{M^{\prime}}_{crx}}/{\alpha _x}l_{{01}}^{2} \hfill \\ {q_{cry}}={{M^{\prime}}_{cry}}/{\alpha _y}l_{{01}}^{2} \hfill \\ \end{gathered} \right.$$

Where L01 is the calculated length in the short span direction; $\alpha_{x},\alpha_{y}$ is the bending moment coefficient in *x* and *y* directions.

After calculation, it is finally obtained that the cracking load of the composite slab is 20.57kN/m^2^. The comparison with the results of finite element simulation is shown in Table 3. The difference between the theoretical calculation value and the finite element results is 11.5% respectively.

### Calculation of ultimate bearing capacity

With the increasing load, when the concrete in the tensile area of the composite slab cracks, the bending moment will be redistributed and concentrated on the reinforcement mesh at the bottom because the concrete here has stopped working. With the increase of load, the tensile reinforcement at the maximum bending moment of the composite slab will gradually yield, and the two-way slab will have a great moment redistribution. When loaded again, the serious crack line will start from the point at which it first yields until there are enough points to form a plastic hinge line and divide the plate into several parts. Once this failure mechanism is formed, the two-way plate can not bear greater load^27^.

**Fig. 20:**
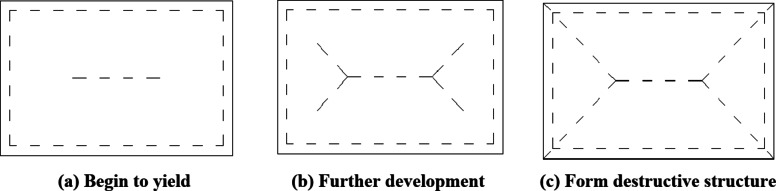
The development process of plastic hinge line of composite floor slab.

For the laminated two-way slab simply supported on four sides, the first crack generally appears in the middle of the slab bottom, parallel to the long span direction of the slab, and the subsequent crack expands in the direction of about 45 ° with the slab edge until the reinforcement at the crack at the slab bottom yields and forms a plastic hinge line on the slab surface. The process is shown in Fig. 20^28^.

According to the basic principle of virtual work, under the action of the same force system, the rigid body in equilibrium produces a small virtual displacement, and the sum of virtual work done by all forces in the force system is equal to zero, that is, the work done by the external force of the component is equal to the work done by the internal force^29^.

**Fig. 21:**
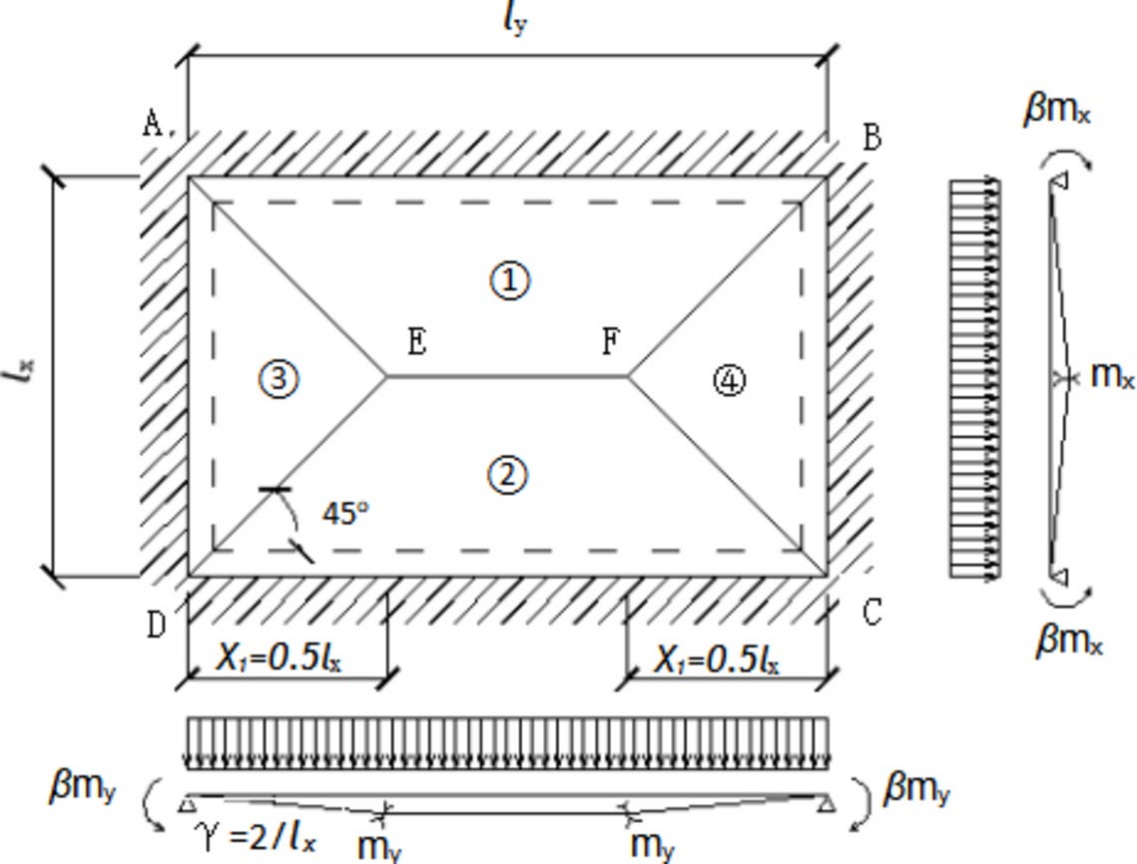
The stress diagram of simply supported composite slab.

Figure 21 shows a simply supported two-way slab on four sides under uniformly distributed load, with *l*_*x*_ on the short side and *l*_*y*_ on the long side, and set *n = l*_*y*_*/l*_*x*_; *m*_*x*_ and *m*_*y*_ are the unit ultimate bending moment of the mid span slab along the short span direction and the long span direction respectively, let *my = kmx*, where:

8 $${m_x}={A_{sx}}{f_y}{\omega _s}{h_{ox}}$$

9 $${m_y}={A_{sy}}{f_y}{\omega _s}{h_{oy}}$$

Where *A*_*sx*_*,A*_*sy*_*,ω*_*s*_*h*_*0x*_*,ω*_*s*_*h*_*0y*_ are the cross-sectional area of longitudinal stressed reinforcement and its internal force coupling arm of slab span belt with unit width along the X and Y directions. *ω*_*s*_ is the internal force arm coefficient, which can be taken as 0.95 in general.

The mid span EF produces unit virtual displacement, and the displacement angle between plates is θ, According to the principle of virtual work, the sum of virtual work made by external load and internal force is equal to zero. The work done by external force can be calculated by the volume of ABCDEF:

10 $$W=q \times \left[ {\frac{{\text{1}}}{{\text{3}}}{l^2}{}_{x}+l{}_{x} \times \left( {l{}_{y} - l{}_{x}} \right) \times \frac{{\text{1}}}{{\text{2}}}} \right]=\frac{{ql_{x}^{2}}}{6}\left( {3n - {\text{1}}} \right)$$

The work done by the internal force can be obtained from the work done by the ultimate bending moment on each plastic hinge line at its corresponding corner:

11 $$U= - \sum {{l_i}} {m_i}{\gamma _i}= - 4\left( {2n+k} \right)\left( {1+\beta } \right){m_x}$$

Where *β* Is the position parameter of plastic strand.

From the sum equal to zero, we can obtain:

12 $$q=\frac{{2n - 2k}}{{3n - 1}}\left( {1+\beta } \right)\frac{{12{m_x}}}{{l_{x}^{2}}}$$

The four sides of the test slab are simply supported, so the bending moment of the support is zero, i.e. *β* = 0, the formula can be simplified as:

13 $$q=24\frac{{n - k}}{{3n - {\text{1}}}}\frac{{{m_x}}}{{l_{x}^{2}}}$$

When solving the ultimate bearing capacity of two -way slab, the strength of the reinforcement is calculated according to the ultimate tensile strength, that is, it is considered that the reinforcement has fully yielded and will be destroyed. Finally, the cracking load of the composite slab is 56.34kN/m^2^. The comparison with the finite element simulation results is shown in Table 3. The difference between the theoretical calculation value and the finite element results is 8.3% (DBS-2).

### Deflection calculation

**Fig. 22:**
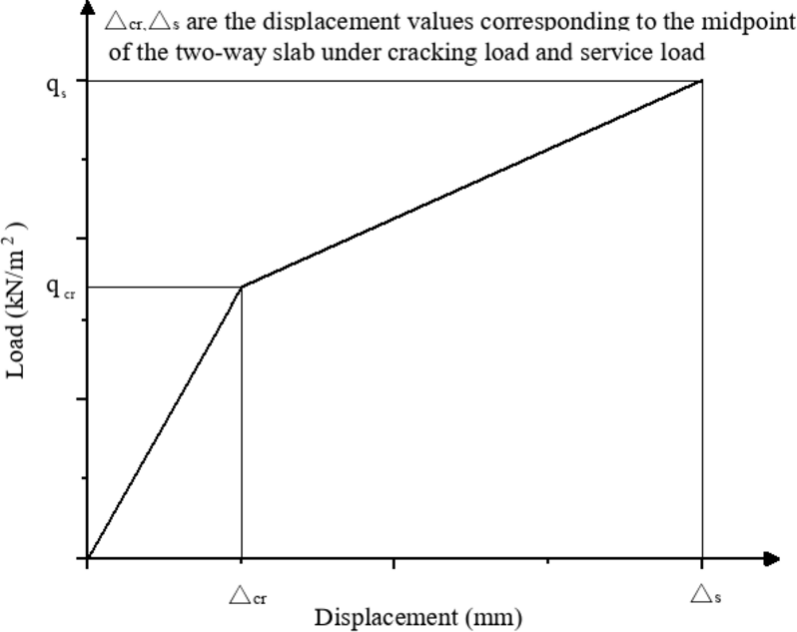
Load-displacement curve of composite floor slab (in the form of two broken lines).

In the process of loading the composite slab to failure, the deflection of the slab does not always change linearly. Therefore, it is necessary to calculate the deflection under the ultimate bearing capacity of the composite slab by sections. In order to facilitate the solution, the load displacement curve of the laminated plate is simplified into bilinear, and the load and deflection at the break point correspond to the cracking of the plate^30^. The calculation shall be divided into two stages: before concrete cracking and after concrete cracking, as shown in Fig. 22.

Before the concrete cracks, the deflection calculation formula of the slab is:

14 $$\Delta =f{q_s}l_{0}^{4}$$

Where, *f* is the deflection coefficient of two-way slab, which is checked according to relevant tables; *q*_*s*_ is the service load and *l*_*0*_ is the length of the shorter side of two -way slab.

After the concrete cracks, the deflection calculation formula of the slab is:

15 $$\Delta =\frac{{f{q_{cr}}l_{0}^{4}}}{{{C_1}}}+\frac{{f\left( {{q_s} - {q_{cr}}} \right)l_{0}^{4}}}{{0.6{C_2}}}$$

*q*_*cr*_ is the cracking constant in formula *C*_*1*_ and *C*_*2*_.

In the uncracked stage, the distance *e* between the neutral axis and the center of the section is required. Since the height *x*_*0*_ of the compression zone has been calculated before, *e = x*_*0*_*-h/2* can be calculated directly, and *η = e/h*, the constant *C*_*1*_ can be obtained, where *ρ = A*_*s*_*/bh*_*0*_ is the reinforcement ratio.

16 $${C_1}=1+12{\eta ^2}+12\eta \rho \frac{{{h_0}}}{h}{\left( {\frac{{{h_0}}}{h} - \frac{1}{2} - \eta } \right)^2}$$

After cracking, the position of the neutral axis is transferred to the lower edge of the compression zone, i.e. *A*_*c*_*=kbh*_*0*_, which is calculated according to the equilibrium equation *A*_*c*_*kh*_*0*_*/2 = n'A*_*s*_*(1-k)h*_*0*_:

17 $$k=\sqrt {{{\left( {n^{\prime}\rho } \right)}^2}+2n^{\prime}\rho } - n^{\prime}\rho$$

Further more

18 $${C_2}={\left( {\frac{{{h_0}}}{h}} \right)^3}\left[ {4{k^3}+12n^{\prime}\rho {{\left( {1 - k} \right)}^2}} \right]$$

Where *n'=E*_*s*_*/E*_*cr*_ is the modulus ratio, *E*_*cr*_*=E*_*s*_.

When composite slab reaches to its ultimate bearing capacity, the deflection is 68.61 mm. The comparison with the simulation results is shown in Table 3. The difference between the theoretical calculation value and the simulation results is 7.5%.

**Table 3:** Theoretical calculation value and FEM results.

	FEM results	Theoretical calculation value
Cracking load(kN)	18.2	20.57
Ultimate bearing capacity(kN)	51.7	56.34
Deflection(mm)	63.48	68.61

While the theoretical model provides a comprehensive framework for calculating the cracking load, ultimate bearing capacity, and deflection of the composite slab, it is important to recognize its limitations. One significant limitation is the assumption of constant material properties, which neglects the degradation of material performance over time due to factors such as creep and shrinkage in concrete. This simplification may lead to discrepancies between theoretical predictions and long-term experimental observations, especially in structures subjected to sustained loading. Additionally, the model assumes homogeneous and isotropic material behavior, which may not fully capture the complexities of real-world composite slabs where material properties can vary.

## Conclusions

Based on the full-scale test, finite element simulation and theoretical calculation of the new type composite floor slab with split joint, this paper explores the static performance of the composite slab from the whole loading process, and obtains the following conclusions:

1. When the slab load is 2kN / m^2^, the vertical deflection of this new type composite slab is 0.103 mm; When the load is 5kN / m^2^, the vertical deflection is 0.481 mm and is in elastic stress state. Explain that it can meet the requirements of design load.
2. The deformation of the new type composite floor slab with split joint shows good integrity, and after reaching the ultimate load, the bearing capacity of the composite slab has not decreased significantly, showing a certain safety reserve.
3. The connecting reinforcement of precast bottom slab is the main stress-bearing member at the joint, and under the same slab uniformly distributed load, the closer the connecting reinforcement to the center area of the slab, the greater the tensile strain. The anti pavement reinforcement at the joint can effectively inhibit the development of cracks in the concrete slab, and the closer it is to the center of the slab, the more obvious the effect of inhibiting cracks is.
4. The theoretical calculation results of the new type composite floor slab with split joint are in good agreement with the simulation results and test results. The calculation method in this paper can provide reference for practical engineering application.
5. The comparative analysis confirms that the finite element simulation effectively captures the mechanical behavior of the new composite floor slab, with results closely matching experimental data. Minor discrepancies in crack width and reinforcement strain suggest areas for model refinement. Overall, the simulation serves as a reliable tool for predicting slab performance and guiding future design and optimization in practical applications.

In summary, the design and application of the new composite floor slab with split joints offer significant advantages for modern construction. Engineers should prioritize centralizing reinforcement to minimize collisions and enhance structural integrity, while precise construction practices, such as controlled slab spacing and reinforcement protection, are crucial for realizing the full benefits of this design. Load testing is essential to confirm the slab’s high bearing capacity and safety reserve, and finite element simulation can further optimize design details. This innovative slab design not only reduces reinforcement usage and minimizes waste but also aligns with economic and sustainability goals, making it an efficient and safe solution for contemporary building projects.

From a practical standpoint, the findings suggest that current design codes may need to be updated to incorporate the benefits of split joint designs, particularly in terms of crack control and load transfer efficiency. Future revisions of design codes should consider these enhanced performance characteristics to promote wider adoption of composite slabs in construction projects. Additionally, future research should focus on the long-term performance of composite slabs, including their durability under cyclic loading and the effects of creep. A deeper understanding of these aspects will provide a more comprehensive assessment of the slab’s performance over its service life and contribute to the development of more robust and reliable design guidelines.

## Data Availability

The datasets used and analysed during the current study available from the corresponding author on reasonable request.
